# Engineered therapeutic antibodies with mannose 6-phosphate analogues as a tool to degrade extracellular proteins

**DOI:** 10.3389/fimmu.2024.1273280

**Published:** 2024-03-12

**Authors:** Morgane Daurat, Corentin Gauthier, Khaled El Cheikh, Lamiaa M. A. Ali, Elodie Morère, Nadir Bettache, Magali Gary-Bobo, Alain Morère, Marcel Garcia, Marie Maynadier, Ilaria Basile

**Affiliations:** ^1^ NanoMedSyn, Montpellier, France; ^2^ Institut des Biomolécules Max Mousseron (IBMM), University of Montpellier, CNRS, ENSCM, Montpellier, France; ^3^ Department of Biochemistry Medical Research Institute, University of Alexandria, Alexandria, Egypt

**Keywords:** therapeutic antibodies, targeted protein degradation, mannose 6-phosphate receptor, M6Pn, immune diseases, cancer, inflammatory diseases

## Abstract

Inducing the degradation of pathological soluble antigens could be the key to greatly enhancing the efficacy of therapeutic monoclonal antibodies (mAbs), extensively used in the treatment of autoimmune and inflammatory disorders or cancer. Lysosomal targeting has gained increasing interest in recent years due to its pharmaceutical applications far beyond the treatment of lysosomal diseases, as a way to address proteins to the lysosome for eventual degradation. Mannose 6-phosphonate derivatives (M6Pn), called AMFA, are unique glycovectors that can significantly enhance the cellular internalization of the proteins conjugated to AMFA *via* the cation-independent mannose 6-phosphate receptor (M6PR) pathway. AMFA engineering of mAbs results in the generation of a bifunctional antibody that is designed to bind both the antigen and the M6PR. The improvement of the therapeutic potential by AMFA engineering was investigated using two antibodies directed against soluble antigens: infliximab (IFX), directed against tumor necrosis factor α (TNF-α), and bevacizumab (BVZ), directed against the vascular endothelial growth factor (VEGF). AMFA conjugations to the antibodies were performed either on the oligosaccharidic chains of the antibodies or on the lysine residues. Both conjugations were controlled and reproducible and provided a novel affinity for the M6PR without altering the affinity for the antigen. The grafting of AMFA to mAb increased their cellular uptake through an M6PR-dependent mechanism. The antigens were also 2.6 to 5.7 times more internalized by mAb-AMFA and rapidly degraded in the cells. Additional cell culture studies also proved the significantly higher efficacy of IFX-AMFA and BVZ-AMFA compared to their unconjugated counterparts in inhibiting TNF-α and VEGF activities. Finally, studies in a zebrafish embryo model of angiogenesis and in xenografted chick embryos showed that BVZ-AMFA was more effective than BVZ in reducing angiogenesis. These results demonstrate that AMFA grafting induces the degradation of soluble antigens and a significant increase in the therapeutic efficacy. Engineering with mannose 6-phosphate analogues has the potential to develop a new class of antibodies for autoimmune and inflammatory diseases.

## 1 Introduction

Monoclonal antibodies (mAbs) represent an important class of innovative therapeutics with more than one hundred antibodies approved or under regulatory review by the FDA and EMA (1). They are mostly applied in the treatment of cancer, autoimmune or inflammatory diseases (2). The targets of mAbs can be classified into two groups: membrane and soluble antigens. Concerning soluble targets, mAbs exert their therapeutic effect primarily by binding to the antigen, resulting in the neutralization of its functions. However, this phenomenon could increase the concentration of the antigen above baseline because of the accumulation of the antibody-antigen complex in the plasma (3). Therapeutic antibodies block the physiopathological function of soluble antigens, although mAbs have limited effect on their expression level and degradation.

Targeted protein degradation (TPD) emerged in 1999 as a key strategy to improve therapeutic efficacy (4). Among TPD approaches, Proteolysis Targeting Chimeras (PROTACs) offer a promising strategy to eliminate intracellular proteins implicated in the pathology *via* the ubiquitin-proteasome system (5–7). PROTAC advances led to the development of autophagy-targeting chimeras (AUTACs) and autophagosome-tethering compounds (ATTECs), all limited to intracellular target proteins (8). More recently, PROTAC technology has been extended to antibody-based PROTACs (AbTACs) for intercellular TPD, notably with bispecific antibodies using the endo-lysosomal pathway as an alternative physiological degradation route (9).

Another way to achieve the antigen degradation is through the lysosomes, which are the main cellular compartments for protein degradation. Lysosomal addressing can be obtained *via* two mannose 6-phosphate receptors, the cation-dependent or the cation-independent receptor, which constitute the major delivery pathways for the lysosomal enzymes. The cation-independent mannose 6-phosphate receptor (here named M6PR) also allows the endocytosis of proteins carrying the mannose 6-phosphate (M6P) signal (10). Different strategies have been developed to improve the targeting of M6PR. Proteins have been modified to express high mannose 6-phosphate oligosaccharidic chains that are still degraded by plasmatic phosphatases (11).

Synthetic analogues of the mannose 6-phosphate were designed to overcome phosphatases degradation (12, 13) and subsequently improved analogues were developed (14) to address extracellular recombinant enzymes to the lysosomal compartment. These analogues, known as analogues of M6P functionalized at anomeric position (AMFA), proved to be a valid solution for an efficient lysosomal targeting. AMFA were the first M6P analogues to be successfully grafted to a recombinant protein and their conjugation to a lysosomal enzyme such as the acid alpha- glucosidase (rhGAA) conferred to the enzyme an improved internalization ability compared to the natural M6P-bearing rhGAA and an increased therapeutic efficacy (14–16). The AMFA analogues were also the basis for the development, in 2020, of large polyglycopeptides containing 20-90 units of the mannose 6-phosphonate by Banik SM. et al. to be grafted to therapeutic antibodies directed against membrane proteins. These complexes, called Lysosome-Targeting Chimeras (LYTACs), are meant to address targeted proteins to the endo-lysosomal pathway (17).

In this study, we explore the application of the original AMFA technology for the conjugation to therapeutic antibodies directed against two different soluble antigens, in the aim of degrading these soluble proteins that are involved in the pathological evolution of various diseases.

The tumor necrosis factor-α (TNF-α), a pleiotropic cytokine implicated in various conditions such as infections, malignancies, and inflammatory/autoimmune diseases, and the vascular endothelial growth factor (VEGF), a key regulator of physiological and tumor angiogenesis, were chosen as the soluble proteins to be targeted for degradation. Although TNF-α is not detectable in healthy individuals, its levels drastically rise in inflammatory and infectious conditions. TNF-α is mainly produced by monocytes and macrophages during inflammatory diseases. Infliximab (IFX) is a human–murine chimeric IgG1 monoclonal antibody directed against TNF-α that has been selected for AMFA-grafting (18). IFX was the first TNF-α inhibitor and the first antibody to receive FDA approval for the treatment of Crohn’s disease in 1998. It was later approved for other adult and pediatric indications in immune-mediated inflammatory conditions (19). Currently, IFX is used to treat rheumatoid arthritis, ankylosing spondylitis and psoriatic arthritis. In terms of VEGF targeting, bevacizumab (BVZ) has been selected for this study. BVZ was the first VEGF inhibitor approved for the treatment of metastatic colorectal cancer. Its indications now include glioblastoma, renal cell carcinoma, cervical cancer, ovarian cancer, non-small cell lung cancer and metastatic breast cancer.

In a recent study, we demonstrated that AMFA specific grafting did not impact the ability of the mAb to bind to the neonatal Fc Receptor (FcRn), a major actor in the recycling of immunoglobulins which is necessary for maintaining suitable plasmatic levels of the antibody. AMFA grafting onto the antibody was stable for weeks *in vivo* and did not induce a modification in the half-life of the antibody in mice, one of the most critical aspects of therapeutic antibodies (20).

In the present work, the AMFA-engineering of the two antibodies, IFX and BVZ, was found to increase the cell uptake of the antigens through the M6PR pathway and to induce their degradation in different cell lines. This degradation of the antigen was associated with a significant increase in the efficacy of the antibodies in cell cultures and in zebrafish and chick embryo models of angiogenesis.

## 2 Materials and methods

### 2.1 Materials

Cell culture media and supplements were provided by Sigma-Aldrich, Fisher Scientific and Promocell. Chemicals and other reagents were supplied by Sigma Aldrich, Fisher Scientific and Abcam. AMFA1 was synthetized as previously described (14). Infliximab is marketed as Remicade^®^ (Janssen Biotech, Inc.) and bevacizumab from Selleckchem and Samsung Bioepis was used for BVZ experiments.

### 2.2 AMFA2 synthesis

The AMFA2 was developed for the grafting of AMFA to the lysines. The synthesis of AMFA2 was based on a previously described method (14) by modifying the spacer arm, replacing the oxyamine function located on the linker with an amine function. Subsequently, the amine function reacts with diethylsquarate ester to generate AMFA2 which can be easily grafted to the ε-amine of lysine residues. Details of the synthesis are presented in **Supplementary Material S1**.

### 2.3 Cell culture

Human T-lymphocyte Jurkat cell line and monocytic THP-1 cells were maintained in RPMI 1640 GlutaMax culture medium supplemented with 10% fetal bovine serum (FBS) and 1% penicillin-streptomycin (P/S). L929 mouse fibroblasts were cultured in Dulbecco’s Modified Eagle Medium (DMEM) supplement with 5% FBS and 1% P/S. Human umbilical vein endothelial cells (HUVECs) were cultured in Endothelial Growth Medium 2 (EGM2). MDA-MB-231 and MCF-7 breast cancer cells were maintained in DMEM supplemented with 10% FBS and 1% P/S.

### 2.4 Macrophage-like differentiation

For macrophage-like differentiation, cells were plated in 96-well plates and incubated with 100 ng.mL^-1^ of phorbol-12-myristate 13-acetate (PMA) for 48 h. After 24 h at rest, macrophage-like cells were used for microscopy experiments in RPMI 1640 GlutaMax medium supplemented with 10% FBS and 1% P/S.

### 2.5 Chemical conjugation of AMFA and AMFA quantitation per antibody

To perform the grafting of AMFA1 onto the oligomannosidic moiety, the oligosaccharidic chains of the antibody were oxidized with different concentrations of sodium metaperiodate solution at 4°C (1 mM for IFX and 2 mM for BVZ). After 30 min, the oxidation was stopped by the addition of 20 µL.mL^-1^ glycerol and the antibody was purified on a G-25 Sephadex column. AMFA1 was then added to the oxidized antibody and the mixture was incubated at 37°C for 2 h under gentle agitation. The grafted antibody was dialyzed overnight against a phosphate buffer.

A second type of grafting was performed with AMFA2 on the lysine moieties of BVZ by incubating the reagents at 25°C for 24 h under gentle agitation. Two protein/AMFA molar ratios were tested, 1:15 and 1:30. Samples were then dialyzed overnight against a phosphate buffer.

The molecular masses of the antibodies before and after AMFA coupling were determined by MALDI-TOF assays.

### 2.6 mAb-AMFA analysis

After AMFA coupling, non-reducing and reducing SDS-PAGE electrophoreses were carried out on IFX, BVZ, IFX-AMFA1, BVZ-AMFA1 and BVZ-AMFA2. The protein concentrations were quantified by Bradford method, and a fixed amount was resolved and transferred to PVDF membranes. Membranes were probed with horseradish peroxidase (HRP) conjugated secondary anti-human IgG antibody (dilution 1:5,000, Jackson ImmunoResearch) or with rabbit anti-AMFA antibody (dilution 1:1,000, NanoMedSyn) and anti-rabbit HRP-conjugated antibody (1:10,000, Jackson ImmunoResearch) and detected by ECL detection system (Perkin Elmer).

### 2.7 Analysis of AMFA position onto IFX

IFX-AMFA1, from a batch prepared at a lower oxidation condition and resulting into 1 AMFA grafting, and IFX were incubated with papain, a sulfhydryl protease, for a proteolytic cleavage. The incubation of papain with IFX and IFX-AMFA1 substrates was conducted at a ratio of 1:3 (w/w) enzyme to mAb, at 37°C for 24 h in the presence of 10 mM L-cysteine. After the digestion process, the antibody samples were subjected to MALDI-TOF-MS analysis in the positive mode using sinapic acid as matrix.

### 2.8 mAb-AMFA affinity for the antigen

The respective antigens, TNF-α for IFX or VEGF for BVZ, were adsorbed on a Maxisorp plate in carbonate buffer pH 9.6 overnight at 4°C. After saturating the plate with 2% BSA, the samples containing IFX, IFX-AMFA1, or BVZ, BVZ-AMFA1 and BVZ-AMFA2 grafted with 5 AMFA (1:15 molar ratio for grafting reaction), here defined as BVZ-AMFA2_(n=5),_ and 16 AMFA (1:30 molar ratio), here defined as BVZ-AMFA2_(n=16),_ were incubated at different concentrations in TNF-α or VEGF coated plates, respectively, for 2 h at room temperature. Finally, after 4 washes, the level of mAb binding to the antigen was determined by incubating the samples with an anti-human IgG HRP-conjugated antibody (1:5,000) and further addition of o-phenylenediamine dihydrochloride (OPD) substrate. The absorbance was read at 450 nm.

### 2.9 mAb-AMFA affinity for M6PR

The affinity of BVZ-AMFA for M6PR was tested by an ELISA method. M6PR (0.5 µg) was adsorbed on a Maxisorp microplate overnight at 4°C. After saturating the plate with 2% BSA, antibody concentrations ranging from 5.10^-8^ to 5.10^-6^ M were incubated at 37°C for 90 min. The antibodies bound to the adsorbed M6PR were detected by incubation with an anti-human IgG HRP-conjugated antibody (1:5,000) and further addition of 3,3’,5,5’-tetramethylbenzidine (TMB) substrate. The absorbance was read at 650 nm.

### 2.10 Internalization of IFX-AMFA1 in cells by flow cytometry, confocal microscopy and western blotting

Flow cytometry experiments were performed on human T-lymphocyte Jurkat cells to assess the internalization of IFX-AMFA1 compared to IFX. Both antibodies were grafted with AlexaFluor647^®^ dye according to the manufacturer’s protocol and cells were treated with 0.75 µg.mL^-1^ IFX or IFX-AMFA1 and 0.188 µg.mL^-1^ TNF-α for 5, 17 or 24 h. To evidence the role of M6PR in the mAb-AMFA cell uptake, an AMFA excess (6.08 mg.mL^-1^) was added to the medium for saturating M6PR and thus avoiding the binding of mAb-AMFA. Then, cell fluorescence measurements were performed by flow cytometry to assess the internalization of mAb or mAb-AMFA.

Internalization of IFX and IFX-AMFA1 in cells was assessed by confocal microscopy on macrophage-like cells that were treated with 0.75 µg.mL^-1^ IFX or IFX-AMFA1, previously labeled with AlexaFluor647^®^ dye, for 5 h. Images were acquired with the ZEISS LSM880 FastAiryscan confocal microscope. Quantification of microscopy images was performed by measuring fluorescence intensity of the antibodies using ImageJ software on 6-7 images per condition, consisting of an average of 100 cells.

### 2.11 Internalization of BVZ, BVZ-AMFA2_(n=5)_ and VEGF in MDA-MB-231 cells by western blotting

MDA-MB-231 cells were plated at 800,000 cells/well in a 6-well plate. Cells were treated with 10 ng.mL^-1^ BVZ, BVZ-AMFA2_(n=5)_ in the presence of 5 ng.mL^-1^ VEGF for 24 h. After treatment, cell lysates and supernatants were subjected to Western blot analysis. After blotting, the gel was transferred onto PVDF membrane then VEGF and mAbs were detected by probing with anti-VEGF rabbit antibody (dilution 1:500, Invitrogen) followed by anti-rabbit HRP-conjugated antibody (1:10,000, Jackson ImmunoResearch) or anti-human HRP-conjugated antibody (1:5,000, Jackson ImmunoResearch), respectively. Membranes were revealed by ECL detection system (Perkin Elmer). Actin was used as a loading control. Membranes were probed with anti-actin antibody (1:1,000, Abcam) and anti-rabbit HRP-conjugated antibody (1:10,000, Jackson ImmunoResearch) followed by ECL detection.

### 2.12 Degradation of the TNF-α in Jurkat and THP-1 cells

To evaluate the degradation of TNF-α in cells, human T lymphocyte Jurkat cells were treated with 5 ng.mL^-1^ TNF-α and 20 ng.mL^-1^ IFX or IFX-AMFA1 for 1, 5, 17 and 24 h. After treatment, cell lysates and supernatants were analyzed by Western blot to assess the TNF-α level. Membranes were probed with anti-hTNF-α rabbit antibody (dilution 1:500, Peprotech) and anti-rabbit HRP-conjugated antibody (1:10,000, Jackson ImmunoResearch) and analyzed with ECL detection system (Perkin Elmer). Actin was used as a loading control. Membranes were probed with anti-actin antibody (dilution 1:1,000, Abcam) and anti-rabbit HRP-conjugated antibody (1:10,000, Jackson ImmunoResearch) followed by ECL detection.

To demonstrate that the TNF-α is degraded *via* a lysosomal pathway, Jurkat cells were treated with 5 ng.mL^-1^ TNF-α and 20 ng.mL^-1^ IFX-AMFA, and 10 mM of the lysosomal inhibitor NH_4_Cl for 24 h. Prior to the IFX-AMFA treatment, cells were incubated with 10 mM NH_4_Cl for 1 h. After treatment, cells and supernatants were analyzed by Western blot to detect the TNF-α levels. Membranes were probed with rabbit anti-hTNF-α antibody (1:500, Peprotech) and anti-rabbit HRP-conjugated antibody (1:10,000, Jackson ImmunoResearch) and detected by ECL detection system (Perkin Elmer). GAPDH was used as a loading control, therefore membranes were probed with a mouse anti-GAPDH antibody (1:50,000, Proteintech) followed by an anti-mouse HRP-conjugated antibody (1:5,000, Amersham) and detected by ECL detection system (Perkin Elmer).

A dose-response study was performed to establish the effect of increasing antibody concentrations on TNF-α internalization and degradation. Jurkat cells were treated with 5 ng.mL^-1^ TNF-α and 5 ng.mL^-1^ or 50 ng.mL^-1^ IFX or IFX-AMFA for 5 h or 24 h. After treatment, the supernatants of the cells were analyzed by Western blot to quantify the level of TNF-α. Membranes were probed with rabbit anti-hTNF-α antibody (1:500, Peprotech) and anti-rabbit HRP-conjugated antibody (1:10,000, Jackson ImmunoResearch) and detected by ECL detection system (Perkin Elmer). GAPDH was used as loading control. Membranes were probed with anti-GAPDH antibody (1:50,000, Proteintech) and anti-mouse HRP-conjugated antibody (1:5,000, Amersham) followed by ECL detection.

The degradation of the targeted antigen was also evaluated on endogenous TNF-α. THP-1 cells were stimulated with 1 µg.µL^-1^ lipopolysaccharides (LPS) (O111:B4) for 5 h in order to induce a TNF-α release and then treated with 20 ng.mL^-1^ IgG, IFX or IFX-AMFA for 5 h. After treatment, the supernatants were analyzed by Western blot to quantify the TNF-α levels. Membranes were probed with anti-hTNF-α rabbit antibody (1:500, Peprotech) and anti-rabbit HRP-conjugated antibody (1:10,000, Jackson ImmunoResearch) and detected by ECL detection system (Perkin Elmer). GAPDH was used as loading control. Membranes were probed with GAPDH antibody (1:50,000, Proteintech) and anti-mouse HRP-conjugated antibody (1:5,000, Amersham) followed by ECL detection.

### 2.13 Degradation of VEGF in MCF-7 cells

For the degradation of recombinant human VEGF, MCF-7 cells were plated at 390,000 cells/well in 6-well plates. Cells were treated with 20 ng.mL^-1^ BVZ, BVZ-AMFA1 or BVZ-AMFA2_(n=5)_ in the presence of 2.5 ng.mL^-1^ VEGF for 24 h and 48 h. Then, cell lysates and supernatants were subjected to Western blot analysis. After blotting, the gels were transferred onto PVDF membranes and VEGF was detected by probing with an anti-VEGF rabbit antibody (dilution 1:1000, Proteintech) followed by anti-rabbit HRP-conjugated antibody (1:10,000, Jackson ImmunoResearch). Protein detection was realized by ECL detection system (Perkin Elmer). GAPDH was used as loading control. Membranes were probed with anti-GAPDH antibody (1:50,000, Proteintech) and anti-mouse HRP-conjugated antibody (1:5,000, Amersham) followed by ECL detection.

For the degradation experiments of endogenous natural VEGF, MCF-7 cells were seeded at 500,000 cells/well in 6-well plates in DMEM supplemented with 1% FBS. After 48 h, cells were treated with 20 ng.mL^-1^ BVZ, BVZ-AMFA1 or BVZ-AMFA2_(n=5)_ and incubated for 5 h and 48 h. Cell lysates were analyzed by Western blot to determine VEGF levels. Membranes were probed with anti-VEGF mouse antibody (dilution 1:1,500, Proteintech) and anti-mouse HRP-conjugated antibody (1:5,000, Amersham) followed by ECL detection. GAPDH was used as loading control. Membranes were probed with anti-GAPDH antibody (1:50,000, Proteintech) and anti-mouse HRP-conjugated antibody (1:5,000, Amersham) followed by ECL detection.

### 2.14 Neutralization of the anti-proliferative effect of TNF-α

L929 fibroblasts were plated at 10,000 cells/well in a 96-well plate. After 24 h, cells were incubated with 5 ng.mL^-1^ TNF-α and increasing doses of IFX and IFX-AMFA1 ranging from 0.5 to 20 ng.mL^-1^ for 48 h in DMEM supplemented with 5% human serum, to observe how IFX and IFX-AMFA1 could prevent the cytotoxic effect of TNF-α.

A cell viability assay was then performed by incubating cells with 3-[4,5-dimethylthiazol-2-yl]-2,5 diphenyl tetrazolium bromide (MTT) for 3 h. The absorbance of the dissolved formazan salts was measured at 540 nm on a spectrophotometer.

### 2.15 Neutralization of mitogenic effect of VEGF

HUVECs were plated at 9,000 cells/well in a 96-well plate. Cells were incubated with 50 ng.mL^-1^ VEGF and increasing doses of BVZ and BVZ-AMFA1 ranging from 2 to 200 ng.mL^-1^ for 72 h in M199 0.5% FCS in order to prevent the mitogenic effect of VEGF. A cell viability assay with MTT was then performed as described before.

### 2.16 Angiogenesis study in transgenic Tg(fli1:eGFP) zebrafish model

The transgenic fluorescent zebrafish *Tg(fli1:eGFP)* model was selected for angiogenesis study *in vivo* (21). This zebrafish model expresses enhanced green fluorescent protein (eGFP) under the control of the *fli1* promoter specifically in endothelial cells, allowing the observation of the vascular system. *Tg(fli1:eGFP)* embryos were kindly provided by Aurélien Drouard (IGF, Montpellier, France). Embryos were obtained from pairs of adult fish by natural spawning and raised at 28.5°C in tank water. Embryos and larvae were staged according to the literature (22). All experiments with zebrafish embryos were performed according to the guidelines of the European Community Council Directive 2010/63/EU. *Tg(fli1:eGFP)* zebrafish embryos were injected intravenously at 48 h post fertilization with 15 ng of BVZ (n=11) or BVZ-AMFA2_(n=5)_ (n=7) for treated animals or vehicle alone for control animals (n=4). The embryos were allowed to develop at 28°C. Embryos were observed at 0, 24 and 48 h post-injection under the Zeiss LSM880 FastAiryscan confocal microscope. The area of the subintestinal vessels (SIVs) and the average number of blood vessel segments were quantified using ImageJ software.

To ensure that the effect on angiogenesis was due to the combination of mAb-AMFA and not to the AMFA derivative alone, a non-specific immunoglobulin G was grafted with AMFA1 according to the method previously described and the grafting was confirmed by MALDI-TOF analysis. *Tg(fli1:eGFP)* zebrafish embryos were injected intravenously at 48 h post fertilization with 15 ng of BVZ-AMFA1 (n=11) or BVZ-AMFA2_(n=5)_ (n=11) or IgG-AMFA1 (n=11) for treated animals or vehicle alone for control animals (n=8). The embryos were allowed to develop at 28°C. Embryos were observed at 0 and 48 h post-injection under the EVOS M5000 microscope. The area of the subintestinal vessels (SIVs) was quantified using ImageJ software.

### 2.17 Neo-angiogenesis in the chorioallantoic membrane of chick embryos

Fertilized White Leghorn eggs were incubated at 37.5°C with 50% relative humidity for 9 days. At that moment, the CAM was dropped down by drilling a small hole through the eggshell into the air sac, and a 1 cm^2^ window was cut in the eggshell above the CAM. An inoculum of 1.10^6^ MDA-MB-231 cancer cells was added onto the CAM of each egg and then 24 eggs were randomized into 3 groups. Each group was treated either with 1 mg.kg^-1^ of BVZ or BVZ-AMFA2_(n=5)_ or with vehicle alone (0.016% Tween 20, 0.6% mannitol in PBS) on day 10, 12, 13 and 15. On day 16, a picture of the upper CAM with tumor was taken. The number of blood vessels reaching the tumor area and clearly visible in all images (width > 130 μm) was counted in triplicate to evaluate tumor angiogenesis.

### 2.18 Statistical analysis

Statistical analyses were performed by using GraphPad prism 8.0. Student’s t-test was used to determine statistical differences between the means of *in vitro* conditions. Tukey’s test was used to perform one-way ANOVA and mixed effect analysis for multiple comparisons. A *p*-value of less than 0.05 (*p* < 0.05), 0.01 (*p* < 0.01) or 0.001 (*p* < 0.001) was considered statistically significant.

## 3 Results

### 3.1 Characterization of mAb-AMFA

**Scheme 1:**
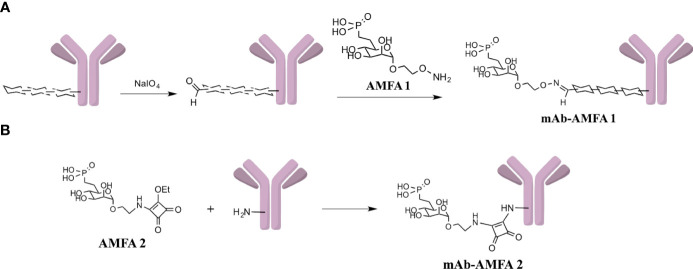
**(A)** Functionalization of oligosaccharidic chains of mAb with AMFA1 leading to mAb-AMFA1 (*i.e.* BVZ-AMFA1 and IFX-AMFA1). **(B)** Functionalization of ε-amine of lysines of mAb with AMFA2 leading to mAb-AMFA2 (*i.e.* BVZ-AMFA2).

**Figure 1:**
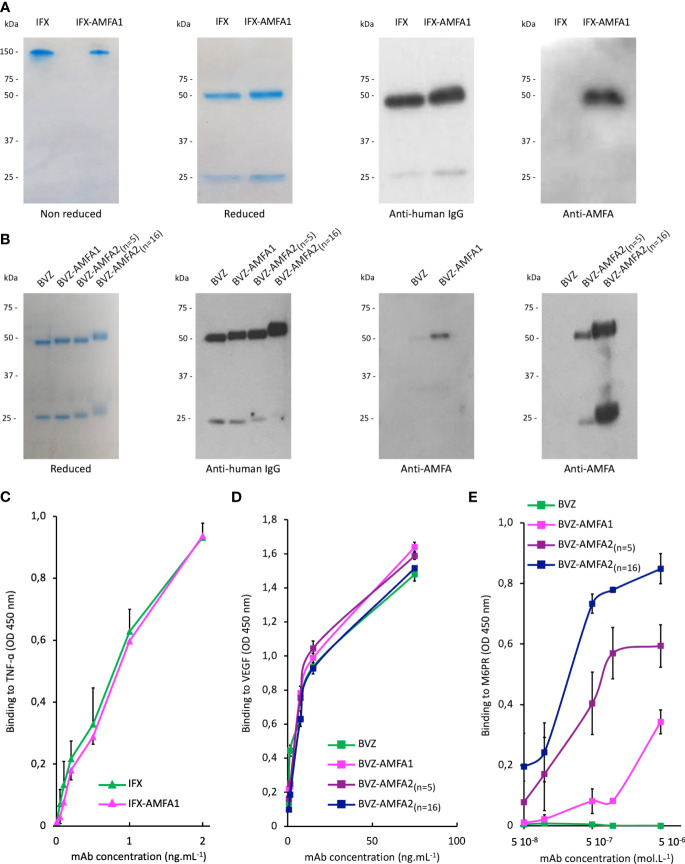
Characterization of Infliximab (IFX) and Bevacizumab (BVZ) before and after AMFA grafting. Analysis of IFX and IFX-AMFA1 **(A)** and BVZ, BVZ-AMFA1 and BVZ-AMFA2 **(B)** under non reducing and reducing conditions of SDS-PAGE followed by Coomassie blue staining or by Western blotting using anti-human IgG or anti-AMFA antibodies. Affinity of IFX and IFX-AMFA1 for TNF-α **(C)**, affinity of BVZ, BVZ-AMFA1 and BVZ-AMFA2 for VEGF **(D)** and the M6PR **(E)**. Binding assays were performed by ELISA method. Results of two independent experiments are presented as mean ± SD of absorbance values at 450 nm.

After conjugation, the number of AMFA grafted per mAb was determined by MALDI-TOF analysis by comparing the masses of the conjugated and non-conjugated mAbs. Two different AMFAs were grafted onto the antibodies. The AMFA1 is a phosphonate analogue that thanks to its ethyloxyamine function at anomeric position allows an oxime ligation onto the oxidized N-glycans of the antibodies. The AMFA2, synthesized as described in **Supplementary Material**, is an analogue designed to be easily grafted onto ε-amine of lysine residues through a squaramide ligation (**Scheme 1**). The results indicated that these AMFAs could be grafted onto IFX or BVZ with high reproducibility standards. Three AMFAs were grafted onto the glycan part of BVZ (3.3 ± 0.6) or IFX (3.3 ± 0.1) using AMFA1, while 5 (5.2 ± 0.2) or 16 AMFA (16 ± 0.2) were grafted on lysines of BVZ-AMFA2 using AMFA2. Moreover, the analyses of the mAb-AMFA by SDS-PAGE showed that AMFA1 or AMFA2 grafting did not induce major protein degradation of the characteristic bands of immunoglobulins under non-reducing or reducing conditions. By using a proprietary anti-AMFA antibody, the AMFA presence on IFX-AMFA1 and BVZ-AMFA1 was detected on the 50 kDa band corresponding to the heavy chain of immunoglobulins (**Figures 1A, B**). In contrast, AMFA2 grafting to the lysine moieties of BVZ was detected on both heavy and light chains (**Figure 1B**). To evaluate in more detail where AMFA1 was grafted, IFX-AMFA1 was digested with papain, a protease that cleaves peptide bonds between the Fc and the Fab fragments. The mass spectral data presented in **Table 1** reveal that the treatment of both IFX and IFX-AMFA1 with papain for 24 h resulted in the complete lysis of the IgG into two Fab fragments observed at m/z 48049 Da and 48036 Da, respectively and two Fc fragments observed at m/z 53392 Da and 53663 Da, respectively, of the heavy chains. Notably, the Fc fragment of IFX-AMFA1 exhibited a higher mass than that of IFX. This mass increase provides evidence that AMFA1 is conjugated onto the Fc fragment of the antibody.

**Table 1:** Analysis of AMFA1 position on IFX after papain treatment.

		Molecular weight (Da)	
		IFX	IFX-AMFA1
Without papain	Whole IgG	150 202	150 563
With papain	Fab fragment	48 049	48 036
	Fc fragment	53 392	53 653

The presence of AMFA on the heavy chains of IFX-AMFA1 was in agreement with the blotting detection of AMFA and with a grafting onto the oligosaccharide moieties carried on the heavy chains, and in particular the *N*-glycosyl chains, as we previously demonstrated thanks to the endonuclease H (PNGase) digestion (20).

Altogether, these results demonstrate the reproducibility of AMFA grafting onto different therapeutic antibodies.

### 3.2 mAb-AMFA affinity for the antigen and M6PR

Antigen binding affinity was also investigated for IFX and BVZ before and after AMFA conjugation. According to ELISA results, the affinity for TNF-α (**Figure 1C**) or VEGF (**Figure 1D**) was not changed after AMFA grafting.

The ability of IFX-AMFA1 to bind to M6PR was validated by a competitive binding inhibition method using a biotinylated M6PR described in our previous studies (20). The affinity of BVZ, BVZ-AMFA1, BVZ-AMFA2_(n=5)_ and BVZ-AMFA2_(n=16)_ for M6PR was investigated by ELISA with adsorbed M6PR and a marked gain in affinity was observed for BVZ-AMFA1, and in particular for BVZ-AMFA2_(n=5)_ and BVZ-AMFA2_(n=16)_, as compared to BVZ. Moreover, the affinity for M6PR increases with the number of AMFA grafted onto BVZ (**Figure 1E**).

### 3.3 Cell uptake of mAb through M6PR pathway

**Figure 2:**
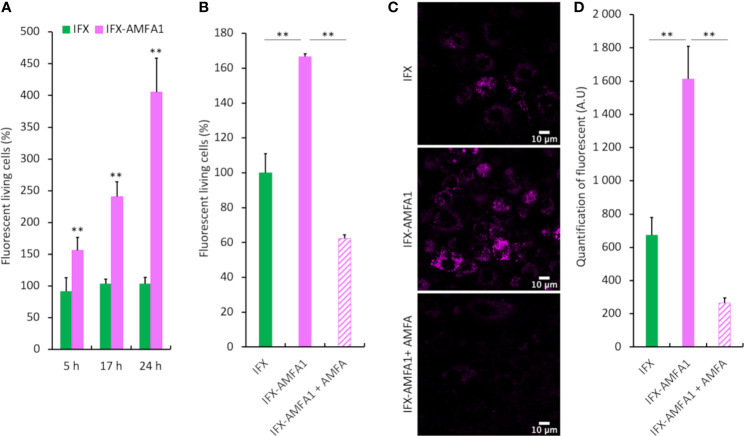
Cellular internalization of mAb and mAb-AMFA in cells and M6PR-dependency. After conjugation with AlexaFluor647^®^ dye, 0.75 µg.mL^-1^ IFX or IFX-AMFA1 were incubated with Jurkat or macrophage-like cells in the presence of 0.188 µg.mL^-1^ TNF-α. Internalization of mAb quantified by fluorescence measurement by flow cytometry at different time points **(A)**. Involvement of M6PR in IFX-AMFA1 endocytosis. An AMFA excess was added to saturate M6PR and the fluorescence was studied by flow cytometry in Jurkat cells **(B)** or by confocal microscopy in macrophage-like cells **(C)**. Quantification of confocal microscopy was performed on an average of 6-7 images consisting each of 100 cells per condition **(D)**. Results are expressed as a percentage ± SD of mAb internalization relatively to IFX **(A, B)** or as mean of intensity ± SD **(D)**. Student’s T-test: ** *p* value < 0.01.

We first needed to determine if IFX-AMFA1 could be more efficiently internalized by cells as compared to mAb using flow cytometry and confocal imaging. At first, IFX and IFX-AMFA1 were equally labelled with AlexaFluor647^®^. Their cellular uptake was then evaluated by fluorescence analysis in human T lymphocyte Jurkat cells treated with 0.75 µg.mL^-1^ labelled IFX and IFX-AMFA1 and in presence of 0.188 µg.mL^-1^ TNF-α for 5, 17 or 24 h. **Figure 2A** shows an increase in IFX-AMFA1 internalization from 5 h to 24 h. At 24 h, IFX-AMFA1 was 3.9-fold more internalized as compared to IFX, used as reference. Moreover, incubation of Jurkat cells with 20 mM AMFA in culture medium to obtain M6PR saturation significantly decreased IFX-AMFA1 internalization hence demonstrating the M6PR involvement in the active endocytosis of IFX-AMFA1 (**Figure 2B**). Complementary data were obtained by confocal imaging. Indeed, a higher fluorescence was detected with IFX-AMFA1 as compared to IFX and only a slight fluorescence was observed for IFX-AMFA1 when an AMFA excess was added (**Figure 2C** and quantitation in **Figure 2**).

The internalization of BVZ and BVZ-AMFA2_(n=5)_ was evaluated in MDA-MB-231 cells treated with 10 ng.mL^1^ BVZ or BVZ-AMFA2_(n=5)_ in the presence of 5 ng.mL^-1^ VEGF for 24 h. **Supplementary Figure S1A** shows a 2.6-fold decrease in BVZ-AMFA2_(n=5)_ level in the supernatants of MDA-MB-231 cells as compared to BVZ demonstrating a high internalization of BVZ-AMFA2_(n=5)_ in cells.

These results clearly demonstrate that a drastic increase in the internalization of IFX-AMFA1 and BVZ-AMFA2 is observed in treated cells and the reversion of this increase with an excess of AMFA clearly indicates the implication of the M6PR pathway.

### 3.4 Internalization of the antigens by mAb-AMFA and degradation

**Figure 3:**
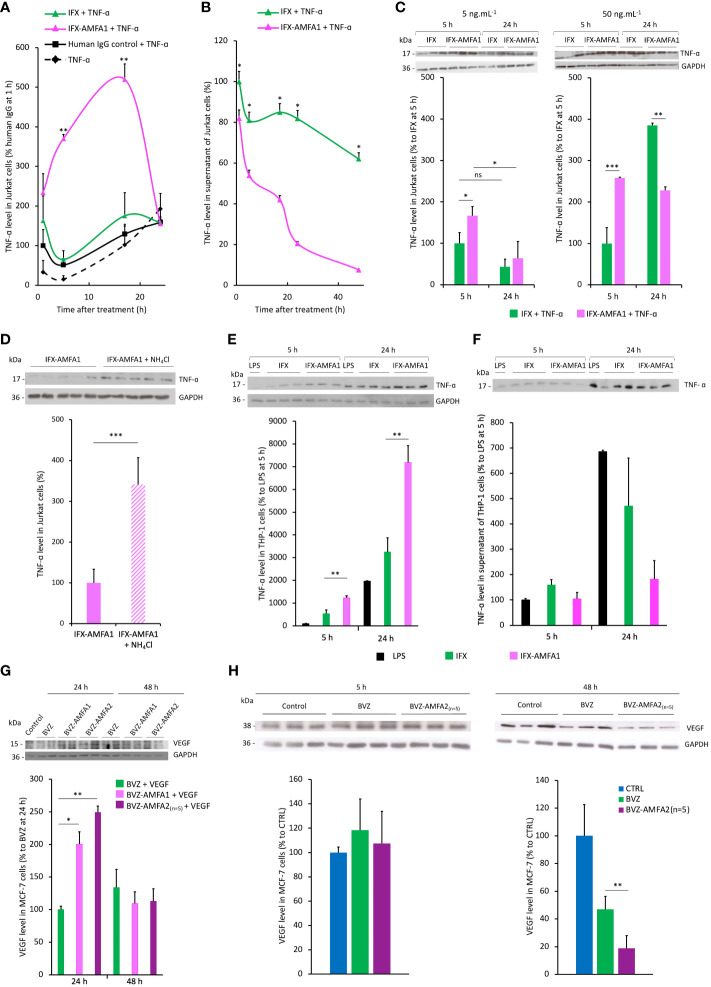
Internalization and degradation of TNF-α and VEGF in cell lines. Internalization in Jurkat cells treated with 20 ng.mL^-1^ IFX, IFX-AMFA1 or human IgG control in the presence of 5 ng.mL^-1^ TNF-α at different time points. Antigen levels in the cell lysates **(A)** or the supernatants **(B)** were analyzed by Western blotting and quantified using the ImageJ software. Original Western blots are presented in **Supplementary Figures S2A** and **B**. Results are expressed as a percentage ± SD of TNF-α internalization relative to human IgG control **(A)**, IFX **(B)** after 1 h. **(A, B)**. Student’s T test: *p* value < 0.05 for IFX-AMFA1 5 h vs IFX-AMFA1 24 h. Dose-response experiment performed in Jurkat cells to demonstrate the efficacy of IFX-AMFA1 in degrading TNF-α as a function of antibody concentration **(C)**. Jurkat cells were treated in triplicate with 5 ng.mL^-1^ TNF-α and 5 ng.mL^-1^ or 50 ng.mL^-1^ IFX or IFX-AMFA1 for 5 h or 24 h. Results are expressed as the percentage of TNF-α in cells as a function of the time of treatment ± SD of IFX-AMFA1-treated cells compared to IFX-treated cells 5 h. Lysosomal involvement in the degradation mediated by IFX-AMFA **(D)**. Jurkat cells were treated with 20 ng.mL^-1^ IFX-AMFA1 and 5 ng.mL^-1^ TNF-α for 24 h in the presence or absence of the lysosomal inhibitor NH_4_Cl (10 mM). Antigen levels in the cell lysates **(D)** or the supernatants (**Supplementary Figure S2C**) were analyzed by Western blotting and quantified using the ImageJ software. Degradation of TNF-α secreted by stimulated THP-1 cells **(E, F)**. TNF-α internalization was assayed on THP-1 cells stimulated with LPS for 5 h and then treated with 20 ng.mL^-1^ IFX or IFX-AMFA1 for 5 h or 24 h. Results represent the mean of triplicates of one representative experiment out of three and are expressed as the percentage of TNF-α in cells **(E)** or in the supernatant **(F)** of these cells ± SD compared to control cells without mAb treatment (n=3). Degradation of VEGF in MCF-7 cells **(G, H)**. MCF-7 cells were treated with 20 ng.mL^-1^ BVZ, BVZ-AMFA1 or BVZ-AMFA2_(n=5)_ and 2.5 ng.mL^-1^ recombinant human VEGF for 24 h or 48 h. VEGF levels in the cell lysates were analyzed by Western blotting. Results represent the mean of duplicates of one representative experiment out of two expressed as a percentage ± SD of VEGF internalization relative to BVZ **(G)**. MCF-7 were maintained in culture medium with 1% FBS for 48 h and produced VEGF, then cells were treated with 20 ng.mL^-1^ BVZ or BVZ-AMFA2_(n=5)_ for 5 h or 48 h. VEGF levels in the cell lysates were analyzed by Western blotting. Results represent the mean of duplicates of one representative experiment out of two as a percentage ± SD of VEGF internalization relative to CTRL **(H)**. Student’s T-test: ns, not significant, * *p* value < 0.05; ** *p* value < 0.01; *** *p* value < 0.001.

The internalization and degradation of TNF-α was evaluated in Jurkat cells treated with 20 ng.mL^-1^ IFX or IFX-AMFA1 in the presence of 5 ng.mL^-1^ TNF-α for 1, 5, 17 and 24 h. The intracellular TNF-α levels were 5.7-fold and 3-fold increased in IFX-AMFA1 treated cells after 5 h and 17 h incubation, respectively, as compared to IFX (**Figure 3A**). However, the amount of intracellular TNF-α drastically decreased after 24 h in IFX-AMFA1 treated cells suggesting its cellular degradation. The degradation of TNF-α by IFX-AMFA1 in **Figure 3B** was also evidenced by analyzing its concentration in the culture medium that indicated a rapid decrease in the antigen level compared to IFX treatment. In order to assess how the therapeutic efficacy of IFX-AMFA1 varies with antibody levels, different concentrations were tested. Interestingly, when a concentration of 5 ng.mL^-1^ IFX or IFX-AMFA1 is used, a 1.7-fold better internalization of TNF-α is found for IFX-AMFA1 compared to IFX although TNF-α levels are inferior to the ones detected at the 20 ng.mL^-1^ mAb concentration previously tested. IFX-AMFA1 already shows a significant degradation of the internalized antigen compared to IFX at 24 h (**Figure 3C**, left panel) and as for the 20 ng.mL^-1^ condition the difference between TNF-α level at 5 h and 24 h is statistically significant for IFX-AMFA1. When a higher concentration (50 ng.mL^-1^) is used, TNF-α is internalized 2.5 times more with IFX-AMFA1 than with IFX after 5 h of treatment. This smaller difference in the internalization rate for IFX-AMFA1 and IFX as compared to that observed at 20 ng.mL^-1^ is probably due to the higher doses of mAb leading to an enhanced passive uptake of IFX. TNF-α accumulates in IFX-treated cells as shown by the significant increase at 24 h of treatment. On the contrary, TNF-α content in IFX-AMFA1-treated cells is significantly lower compared to IFX treatment (**Figure 3C**, **right panel**).

The treatment with the lysosomal inhibitor NH_4_Cl prevented the degradation of TNF-α at 24 h, demonstrating that the protein degradation occurs specifically in lysosomes (**Figure 3D**). **Figure 3D** shows that, after 24 h, cells treated with IFX-AMFA1 together with NH_4_Cl have a significantly higher amount of TNF-α compared to cells only treated with IFX-AMFA alone, which present 3.4 times less antigen. Interestingly, a similar amount of TNF-α is observed in the supernatant of these cells, implying that once internalized by IFX-AMFA1, TNF-α is degraded *via* the lysosomal pathway (**Supplementary Figure S2C**).

To mimic the therapeutic effect of IFX-AMFA1 in the physiological environment, THP-1 cells were stimulated to produce TNF-α and the ability to induce the antigen degradation was evaluated for IFX and IFX-AMFA1.

THP-1 cells were stimulated with LPS for 5 h to induce a secretion of TNF-α. Then, 20 ng.mL^-1^ IFX or IFX-AMFA1 were added to the medium and incubated for 5 and 24 h. In IFX-AMFA1-treated cells, we observe a 2.3-fold higher amount of TNF-α compared to IFX-treated cells at 5 h (**Figure 3E**), although no difference in the cytokine level is observed in the supernatants (**Figure 3F**). At 24 h, a 2.2 fold increase of TNF-α is still observed in the presence of IFX-AMFA1 compared to IFX (**Figure 3E**). It is evident from **Figure 3F** that cell treatment with IFX-AMFA1 allows for a dramatic clearance of TNF-α from the extracellular environment at 24 h. The amount of TNF-α found in the supernatant of IFX-AMFA1-treated cells is 3.7 times lower than in IFX-treated cells, which presented 1.5 times less TNF-α. These results support the ability of the IFX-AMFA1 technology to degrade also the endogenous cytokine.

Regarding BVZ-AMFA efficacy in VEGF degradation, we also observed that its intracellular concentration was initially increased by 2-fold and 2.5-fold in cells treated with VEGF after 24 h incubation with BVZ-AMFA1 and BVZ-AMFA2_(n=5)_, respectively, as compared to BVZ (**Figure 3G**). Thereafter, the level of intracellular VEGF significantly decreased at 48 h in cells treated with BVZ-AMFA1 and BVZ-AMFA2_(n=5)_, following a similar kinetic profile observed for TNF-α and IFX-AMFA1. In contrast, VEGF internalization in BVZ-treated cells was comparable to that in VEGF-treated cells. These data demonstrate that the grafting of the AMFAs to BVZ greatly enhances the cell uptake and the degradation of the antigen in MCF-7 cells. Similar results were obtained in MDA-MB-231 cells at 24 h (**Supplementary Figure S1B**).

To fit at best with BVZ clinical application, we decided to test the degrading effect on the VEGF secreted by cancer cells, in particular MCF-7 breast cancer cells.

Cells were allowed to grow for 48 h and then they were treated with 20 ng.mL^-1^ of BVZ and BVZ-AMFA2_(n=5)_ for 5 and 48 h. While a similar amount of VEGF was observed in cells at 5 h, a significant decrease is detected in the cells treated with BVZ-AMFA2_(n=5)_ at 48 h. Indeed, BVZ-AMFA2_(n=5)_ is able to reduce by 80% the level of VEGF in the treated cells (**Figure 3H**). BVZ-AMFA2_(n=5)_ exhibited a better efficacy on the degradation of secreted VEGF in MCF-7 cells.

These results showed that AMFA grafting granted a better internalization of the circulating antigens followed by a significant reduction in their intracellular levels supporting that AMFA targeting of therapeutic antibodies makes possible the degradation of the extracellular proteins in lysosomes.

### 3.5 Neutralization of the antigen activities on cell proliferation by mAb-AMFA

**Figure 4:**
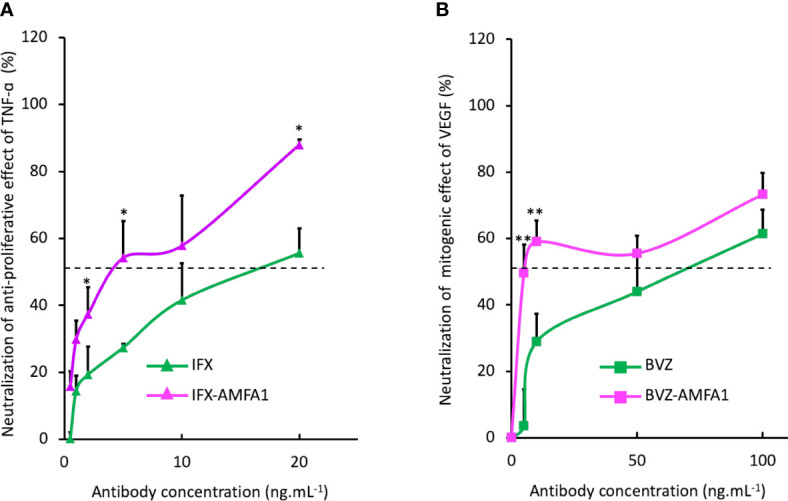
Neutralization of the cellular activities of antigens with mAb-AMFA. Murine fibroblasts L929 were treated with 5 ng.mL^-1^ TNF-α and increasing doses of IFX or IFX-AMFA1 **(A)**. HUVECs were treated with 50 ng.mL^-1^ of VEGF and increasing doses of BVZ or BVZ-AMFA1 **(B)**. The neutralization of antigens effect was evaluated by a cell viability assay by incubating cells with MTT. Results are expressed as a percentage ± SD of control cells without mAb treatment. Student’s T-test: * *p* value < 0.05; ** *p* value < 0.01.

We investigated the IFX-AMFA1 ability to inhibit the biological effect of the TNF-α on murine L929 fibroblasts treated with 5 ng.mL^-1^ TNF-α and increasing doses of IFX or IFX-AMFA1 (0.5 to 20 ng.mL^-1^) for 48 h. Since TNF-α engenders a cytotoxicity on murine L929 fibroblasts, a viability assay was realized to determine the inhibitory effect of IFX and IFX-AMFA1 on the TNF-α response. IFX-AMFA1 induced a dose-dependent neutralization of TNF-α more efficiently than IFX. Moreover, IFX-AMFA1 allowed the neutralization of TNF-α cytotoxicity already at the lowest dose, reaching the IC_50_ at 3.9 ng.mL^-1^ while IFX IC_50_ was reached at 15.8 ng.mL^-1^ (**Figure 4A**). This higher efficacy is consistent with the greater ability of IFX-AMFA1 to internalize and degrade TNF-α in cells. In parallel, the efficacy of BVZ and BVZ-AMFA1 to neutralize the mitogenic effect of VEGF in HUVECs was analyzed by treating cells with 50 ng.mL^-1^ VEGF and increasing doses of BVZ or BVZ-AMFA1 (5 to 100 ng.mL^-1^) for 72 h. Since VEGF is known to induce a proliferation of HUVECs, the number of living cells was quantified after mAb treatments. **Figure 4B** shows that the neutralization of VEGF in HUVECs was more efficient with BVZ-AMFA1 than with BVZ. BVZ-AMFA1 achieved the half maximal response at 5 ng.mL^-1^ while more than 50 ng.mL^-1^ of BVZ were necessary to achieve the same outcome. These data indicate that AMFA grafting to BVZ enables a 10-fold better inhibition of VEGF *in vitro*.

### 3.6 *In vivo* efficacy of BVZ-AMFA in Zebrafish

**Figure 5:**
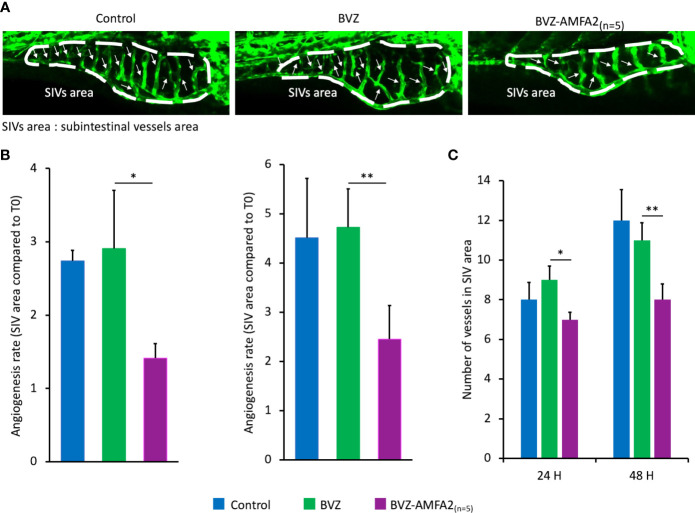
*In vivo* efficacy of BVZ-AMFA2_(n=5)_ in zebrafish angiogenesis model. *Tg(fli1:eGFP)* zebrafish embryos were injected intravenously with 15 ng BVZ or BVZ-AMFA2_(n=5)_ at 48 hours post fertilization. Zebrafish embryos were observed at 0, 24 and 48 h post-injection under confocal microscope. The representative fluorescence images of control (n=8) and embryos treated with BVZ (n=11) or BVZ-AMFA2_(n=5)_ (n=11) at 48 h post-injection are presented for the subintestinal vessels (SIVs) area, and the blood vessels are indicated by arrows **(A)**. The SIVs area **(B)** and the number of blood vessels **(C)** were determined, and the results are expressed as the angiogenesis rate compared to the value at T0 or as the number of blood vessels, respectively. Tukey’s test: * *p* value < 0.05; ** *p* value < 0.01.

The efficacy of BVZ and BVZ-AMFA2_(n=5)_ was investigated *in vivo* by using a specific zebrafish model of angiogenesis. BVZ-AMFA2_(n=5)_ was selected for this study because its affinity for M6PR was shown to be 10-fold higher than that of BVZ-AMFA1 (**Figure 5**). The *Tg(fli1:eGFP)* transgenic line, which is modified in order to express eGFP in endothelial cells and therefore to visualize the blood vessels, was selected for the angiogenesis study (21). More precisely, we focused on the analysis of the subintestinal vessels (SIVs) area and the number of blood vessel segments which are the most commonly used parameters for the study of anti-angiogenic molecules (23). Zebrafish embryos were injected intravenously with 15 ng BVZ (n=11) or BVZ-AMFA2_(n=5)_ (n=7) or vehicle for CTRL (n=4) at 48 h post-fertilization and observed by confocal imaging after the injection and at 24 h and 48 h after treatment. The good angiogenesis development and the eGFP expression of the trunk vessels of zebrafish were controlled (data not shown). **Figure 5A** shows representative fluorescence images of control, BVZ or BVZ-AMFA2_(n=5)_ treated embryos at 48 h post-injection. In control embryos, SIVs area developed normally and the blood vessels were established in an orderly manner and their integrity were maintained in embryos treated with BVZ, no significant difference appeared for the SIVs area and the number of blood vessels compared to control individuals. In contrast, BVZ-AMFA2_(n=5)_ treated embryos showed a significant reduction in SIVs area and a lower number of blood vessels. These results were confirmed by the quantification of SIVs area (**Figure 5B**) and the number of blood vessels (**Figure 5C**) at 24 h and 48 h post-injection by using ImageJ software.

An additional study was performed to confirm that the effect on zebrafish vascularization in the presence of BVZ-AMFA was due to the whole molecule mAb-AMFA and not to the AMFA derivative. Non-specific IgG was grafted with AMFA1 and a number of 7,8 AMFA per molecule was detected. The results presented in **Supplementary Figure S3** show that when AMFA is grafted onto a non-specific IgG, no effect on angiogenesis is observed compared to controls. Interestingly, embryos treated with BVZ-AMFA1 exhibited a reduced SIVs area as with BVZ-AMFA2_(n=5)_ compared to IgG-AMFA1 and untreated individuals.

In conclusion, the AMFA grafting to BVZ allowed a significant decrease in the development of zebrafish vasculature.

### 3.7 *In vivo* efficacy of BVZ-AMFA2_(n=5)_ on tumor angiogenesis in CAM of chick embryos

**Figure 6:**
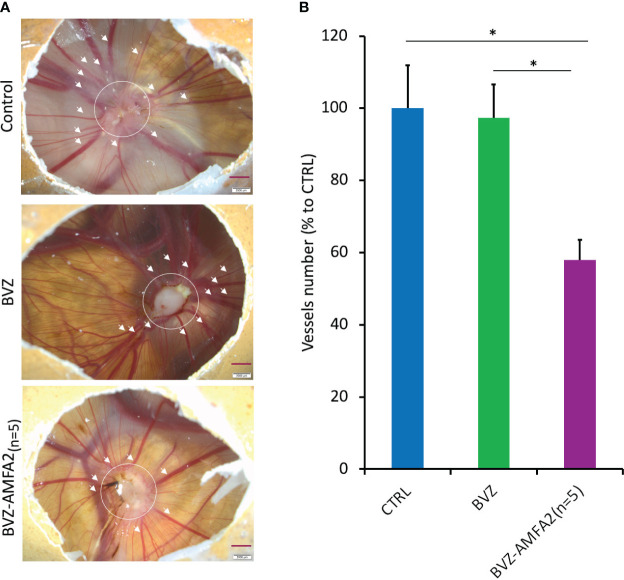
*In vivo* efficacy of BVZ-AMFA2_(n=5)_ tumor angiogenesis in the CAM of chick embryos. After injection of 10^6^ MDA-MB-231 human cancer cells at day 9 in CAM, embryos were treated with 1 mg.kg^-1^ BVZ or BVZ-AMFA2_(n=5)_ or vehicle at days 10, 12, 13 and 15. The representative pictures of control and embryos treated with BVZ or BVZ-AMFA2_(n=5)_ at day 16 are presented and the number of blood vessels of size > 130 µm surrounding the tumor area are indicated by the arrows **(A)**. The mean ± SD of vessels counted in 7 embryos treated with BVZ or BVZ-AMFA2_(n=5)_ and in 7 vehicle-treated embryos are presented **(B)**. Tukey’s test: * *p* value < 0.05.

The angiogenic development around tumors was studied on the CAM of MDA-MB-231 xenografted chick embryos treated with 4 injections of 1 mg.kg^-1^ BVZ or BVZ-AMFA2_(n=5)_ or vehicle (**Figure 6A**). The number of vessels was significantly reduced by the treatment with BVZ-AMFA2_(n=5)_ compared to vehicle and BVZ treatment (**Figure 6B**). In this model of tumor development in CAM, the anti-angiogenic efficacy of BVZ was dependent on the AMFA conjugation to the antibody.

## 4 Discussion

In recent years, PROTACs and, more recently, LYTACs, have emerged as promising approaches to remove targeted disease-associated proteins by exploiting the cellular mechanisms of degradation. Adding this feature to therapeutics could significantly enhance the efficacy of monoclonal therapeutic antibodies targeting soluble antigens, which have gained significant importance in the treatment of numerous diseases (24). In this work, we investigated the effect of the addition of mannose 6-phosphonate analogues, called AMFA, to two therapeutic antibodies, IFX and BVZ, that target soluble antigens. Our objective was to improve the therapeutic effect of these antibodies by taking advantage of M6PR pathway and its ability to deliver proteins to the lysosome for degradation.

We have successfully demonstrated the reproducible conjugation of a controlled number of AMFA derivatives to monoclonal antibodies, and AMFA conjugation was achieved either through an oxime ligation onto the *N*-glycans of the Fc region (20) or through a squaramide ligation to lysine residues. Importantly, the presence of AMFA does not modify the mAb affinity for their targets, even when AMFA is conjugated to lysine residues. Interestingly, AMFA2 grafted onto lysine moieties of BVZ, which may be present in the paratope region, did not modify the mAb affinity for VEGF, even when a higher number of AMFAs were grafted. In addition, AMFA grafting confers mAb an affinity for M6PR and improves the cellular uptake of the mAb through an M6PR-mediated mechanism.

It is well known that the ubiquitous membrane M6PR is the major receptor involved in addressing proteins to the lysosomes. In this context, we have demonstrated that IFX-AMFA1 displayed a significantly higher uptake of TNF-α in human T lymphocyte Jurkat cells as compared to IFX. At the same time, the TNF-α level in the supernatant of Jurkat cells treated with IFX-AMFA1 drastically diminished, demonstrating that AMFA grafting allowed a higher internalization of TNF-α, and induced an important decrease in the TNF-α intracellular level 24 h after treatment. This mechanism is confirmed to depend on the lysosomal pathway as shown by data obtained in the presence of the lysosomal inhibitor NH_4_Cl, which prevented the degradation of the intracellular TNF-α. A better internalization leading to antigen degradation is shown at different doses of IFX-AMFA1, ranging from 5 to 50 ng.mL^-1^, compared to IFX. Enhanced internalization and clearance from the extracellular environment is also described for secreted TNF-α in stimulated THP-1 cells with IFX-AMFA1.

AMFA engineering also managed to improve the efficacy of BVZ. We have demonstrated a significant increase in the internalization of recombinant VEGF with BVZ-AMFA treatment compared to BVZ at 24 h and a decrease in intracellular VEGF levels at 48 h in MCF-7 cells. Similar results were obtained for the degradation of the VEGF secreted by MCF-7 cells by the treatment with BVZ-AMFA2_(n=5)_.

These results demonstrate the ability of mAb-AMFA to internalize and degrade soluble antigens in a more physiological environment corresponding to inflammatory diseases or cancer.

The soluble antigens such as TNF-α and VEGF can have deleterious biological effects associated with various diseases such as inflammatory diseases and cancer. TNF-α induces cell cytotoxicity in fibroblasts while VEGF has a mitogenic effect on endothelial cells implicated in tumor angiogenesis. We have demonstrated that IFX-AMFA1 or BVZ-AMFA1 inhibit the cytotoxicity induced by TNF-α and the mitogenic effect of VEGF on cells, respectively. These results support the ability of mAb-AMFA conjugates to improve their therapeutic potential *in vitro*.

This therapeutic improvement was supported by the *in vivo* studies realized in two different models. The study performed on the *Danio rerio* angiogenesis model provides additional evidence of the efficacy of the mAb BVZ-AMFA2 in reducing the extracellular levels of the targeted soluble antigen (VEGF) and enhancing the therapeutic effect of the mAb. These findings indicate that BVZ-AMFA2 demonstrated a superior efficiency in inhibiting the embryo angiogenesis than BVZ. Moreover, the study in a CAM model of tumor angiogenesis shows a significant anti-angiogenic effect of BVZ only when grafted with AMFA. Based on these results, a higher therapeutic response can be expected with BVZ-AMFA2 compared to BVZ in the context of tumor neo-angiogenesis, leading to a reduction in tumor growth.

Previous studies have reported that despite clinical and biochemical improvement in patients treated with TNF-α inhibitors, the level of circulating TNF-α does not decrease. This observation suggests that the persistence of joint pain in these patients may be due to the presence of circulating TNF-α (25–27). Disappointing results of BVZ trials in advanced prostate, pancreatic and breast cancer have highlighted the importance of an improved treatment that could ensure a better beneficial effect (28). Therefore, a technology that would allow the internalization through the M6PR pathway and the degradation of the targeted antigen is emerging as a real therapeutic option. Recently, Banik et al. described the use of large polyglycopeptides conjugated to the targeting antibodies (LYTACs) for the internalization of membrane antigens *via* the M6PR (17). These glycopeptides are composed of numerous units of alanine-linked mannose 6-phosphonate analogues ranging from 20 to 90, for the M6PR addressing of the antibodies. Although a positive *in vitro* effect on target degradation has been described, little is known about the *in vivo* response and a possible immunogenic issue can be foreseen. A study about recombinant hemagglutinin (HA) glycoprotein-based vaccines evidenced a significantly stronger immunogenic response for a high-mannose (HA) than for a 5 mannose HA (29). A limited and mastered glycosylation rate as the one proposed by the AMFA technology could limit the immunogenic side effects. Furthermore, previous experiences of an AMFA-conjugated protein showed that AMFA grafting is safe and does not induce an immunogenic response, even after several months of treatment (16).

Previous studies have shown that the addition of AMFA does not alter the ability of the antibody to be recycled by the FcRn, thereby preserving its half-life in the bloodstream (20). Accordingly, AMFAs add a new property to mAbs, without modifying this major intrinsic property. Although mAb-AMFAs acquire the potential to bind to M6PR, the affinity for the antigens is still greater than that for M6PR. This suggests that mAbs first and preferably bind to their antigens and then, binding to M6PR would occur, hence leading to the internalization of the mAb-AMFA-antigen complex. While conventional antibodies neutralize their antigens in the bloodstream, AMFAs allow the internalization of the mAb-antigen complex in the cells, thereby improving the therapeutic efficacy.

However, another drawback of conventional antibodies could arise from the stability of the mAb-antigen complex at endosomal pH as well, which would not dissociate in the endo-lysosomal compartment resulting in its accumulation in the cells and finally the degradation in lysosomes. Improvements in antibody engineering have led to the development of pH-sensitive antibodies, that are meant to bind to the antigen with high affinity at physiological pH and release it at the lower pH of the endosomal compartment, hence ensuring the recycling of the antibody and the antigen degradation in lysosomes (30). Therefore, it would also be interesting to study the AMFA conjugation to such pH-sensitive antibodies.

In conclusion, AMFA-engineered antibodies for extracellular protein degradation have been proposed as a novel technology to internalize and degrade soluble antigens through the M6PR pathway. AMFA technology enables the M6PR targeting and preserves the intrinsic properties of therapeutic antibodies that are essential, such as the affinity for their targets, the recycling *via* FcRn and the plasma half-life (20). AMFA grafting on either the oligosaccharidic or aminic moiety has led to a therapeutic improvement. The addition of AMFA to IFX and BVZ allowed an increase in the therapeutic efficacy, *in vitro* and *in vivo.* Furthermore, the potential applications of AMFA-engineered antibodies are not limited to soluble antigens, and ongoing studies are focusing on the degradation of membrane proteins involved in various diseases to fully explore the potential of AMFA analogues.

## Data availability statement

The original contributions presented in the study are included in the article/**Supplementary Material**, further inquiries can be directed to the corresponding author/s.

## Ethics statement

Ethical approval was not required for the studies on humans in accordance with the local legislation and institutional requirements because only commercially available established cell lines were used. Ethical approval was not required for the study involving animals in accordance with the local legislation and institutional requirements because using chick embryos and zebrafish embryos under 5d post fertilization does not require ethical approval.

## Author contributions

MD: Conceptualization, Investigation, Writing – original draft, Writing – review & editing, Formal analysis, Visualization. CG: Conceptualization, Formal analysis, Investigation, Visualization, Writing – original draft, Writing – review & editing. KEC: Investigation, Writing – original draft, Writing – review & editing, Validation. LMAA: Investigation, Visualization, Writing – original draft. EM: Investigation, Writing – original draft, Resources. NB: Writing – original draft, Supervision. MG-B: Supervision, Writing – original draft. AM: Supervision, Writing – original draft, Validation, Writing – review & editing. MG: Supervision, Validation, Writing – original draft, Writing – review & editing, Conceptualization, Project administration. MM: Conceptualization, Supervision, Writing – original draft, Writing – review & editing, Investigation. IB: Conceptualization, Investigation, Supervision, Writing – original draft, Writing – review & editing, Project administration, Validation.
