# Supplementary material for: Engineered therapeutic antibodies with mannose 6-phosphate analogues as a tool to degrade extracellular proteins
Source: Front Immunol. 2024 Mar 12;15:1273280. doi: 10.3389/fimmu.2024.1273280 (PMC10964947; doi:10.3389/fimmu.2024.1273280)
Supplement: Supplementary file 1 [file DataSheet_1.docx]

Supplementary Material

# Supplementary Data: AMF2 synthesis

D-Mannose, reagents and anhydrous solvents were purchased from Sigma-Aldrich or Fisher scientific. Optical rotations were measured using a Perkin Elmer 241 polarimeter (sodium D line: 589 nm). NMR spectra were recorded on Brüker DRX 400 (400 MHz for ^1^H, 101 MHz for ^13^C, and 162 MHz for ^31^P), and Brüker AVANCE III 600 (600 MHz for ^1^H, 150 MHz for ^13^C, and 243 MHz for ^31^P) instruments with Me4Si (δ =0 ppm) as the internal standard. Chemical shifts are reported in ppm, and the following abbreviations were used to explain multiplicities: s = singlet, d = doublet, t = triplet, q = quartet, m = multiplet. All coupling constants are apparent J values measured at the indicated field strengths. Flash column chromatography was carried out using silica gel 60 (230–400 mesh, Merck). Purifications on RP-Gel were performed using reversed-phase silica gel 100 (C_18_, Sigma) columns. ESI MS spectra were measured using a Waters Micromass apparatus. Fluorescence measurements were performed on a Victor 3V spectrophotometer (PerkinElmer).

Scheme S1. Reagents and conditions: (a) 2-bromoethanol, Amberlite IR120, 80 min, 54% (b) TMSCl, Et_3_N, CH_2_Cl_2_, rt, 20h (c) K_2_CO_3_, MeOH, 0°C, 15 min, 60% (d) Dess-Martin periodinane, CH_2_Cl_2_, 1h, rt (e) CH_2_(P(O)(OEt)_2_)_2_, NaH, THF, rt, 1 h, 76% (2 steps) (f) Et_3_SiH, MeOH, Pd/C, 2h, 99% (g) Potassium phthalimide, DMF, 60°C, 22 h, 74%, (h) TMSCl, Et_3_N, CH_2_Cl_2_, rt, 20h, quant. (i) TMSBr, Pyridine, CH_3_CN, rt, 6 h, (j) Hydrazine monohydrate, MeOH, 16h, 65% (2 steps) (k) Diethyl squarate, EtOH/H_2_O, 30 min, rt, 50%.

The synthesis of **AMFA2**, as shown in scheme S1, started by introducing 2-bromoethanol at the anomeric position of commercial D-mannose through a Fischer glycosylation using Amberlite IR120 resin as a catalyst, resulting in the formation of compound **1** with a yield of 54%. Compound **1** was then quantitatively silylated using chlorotrimethylsilane in dichloromethane. The selective deprotection of fully silylated compound **2**, using catalytic amount of K_2_CO_3_ in methanol [4], yielded the trisilylated alcohol **3** (60%). Next, the phosphonate moiety was introduced by oxidizing the trisilylated alcohol **3** to form aldehyde **4**, which subsequently reacted with the tetraethyl methylenediphosphonate anion to produce phosphonate **5** with a yield of 76% over two steps. The reduction of the double bond and the hydrolysis of the trimethylsilyl group on positions 2, 3 and 4 afforded quantitatively compound **6**. The *N*-phthalimido group was then introduced by substituting the bromine atom of **6** with a yield of 74%. The hydroxyl group on positions 2, 3 and 4 were again silylated, leading to the formation of **8** with a quantitative yield. Compound **9** was obtained in 65% yield through the complete deprotection of **8** using the Rabinowitz deprotection of the phosphonate moiety, followed by the hydrazinolysis of phthalimido group to generate the amine function. Finally, the **AMFA2** derivative was synthesized with 50% yield by the reaction with of compound **9** with diethyl squarate.

**2-bromoethyl α-D-mannopyranoside (1)**

D-mannose (15 g, 83.3 mmol) was dissolved in 2-bromoethanol (45 mL, 635 mmol) in the presence of pre-activated Amberlite IR120 resin (15 g). The reaction mixture is heated to 80°C for 3h30, then cooled to room temperature and filtered. The filtrate was evaporated under reduced pressure. The resulting residue (brown liquid) was purified through silica gel chromatography (using CH_2_Cl_2_/MeOH: 0 to 15% MeOH gradient) to obtain compound **1** in the form of a brown liquid with a yield of 54% (12.85g, 44.8 mmol).

R_f_: 0.52 [CH_2_Cl_2_/MeOH (8:2)]

MS, ESI^-^ m/z: 331 [M-H+HCOOH]^-^

^1^H NMR (400 MHz, MeOD, 298K): δ = 3.55 (m, 2H, H-8), 3.60-3.62 (m, 2H), 3.68-3.73 (m, 2H, H-6’ and 1H), 3.78-3.85 (m, 3H, H-6, H-7’ and H-2), 4.01 (m, 1H, H-7), 4.83 (d, *J*_H1-H2_ = 1.7 Hz, 1H, H-1) ppm.

^13^C NMR (101 MHz, MeOD, 298K): δ = 31.5 (1C, C-8), 62.9 (1C, C-6), 68.5 (1C), 68.9 (1C, C-7), 72.0 (1C, C-2), 72.5 (1C), 75.0 (1C), 101.8 (1C, C-1) ppm.

**2-bromoethyl 2,3,4,6-tetra-*O*-trimethylsilyl-α-D-mannopyranoside (2)**

Compound **1** (2 g, 6.97 mmol) was dissolved in CH_2_Cl_2_ (20 mL) and triethylamine (28.2 mL, 209.1 mmol). After addition at 0°C of trimethylsilyl chloride (7.1 mL, 55.76 mmol), the reaction mixture was warmed to room temperature and stirred for 20h. The solvents were then evaporated, and the resulting residue was dissolved in 60 mL of cyclohexane and filtered through celite. The celite was washed with 350 mL of cyclohexane. The cyclohexane was evaporated under reduced pressure to yield compound **2**, which was used for the next step without any purification.

MS, ESI^+^ m/z: 594.2 [M+Na]^+^

R_f_: 0.7 [Cyclohexane/Et_2_O (9:1)]

**2-bromoethyl 2,3,4-tri-*O*-trimethylsilyl-α-D-mannopyranoside (3)**

A methanolic solution of K_2_CO_3_ (80 mL, 0.015 M) was added at 0°C to a stirred solution of compound **2** (17.6 g, 30.65 mmol) in anhydrous methanol (80 mL). After stirring for 15 min at 0°C, the reaction mixture was diluted with 250 mL of CH_2_Cl_2_ and then washed twice with 40 mL of brine. The aqueous layer was extracted with 150 mL of CH_2_Cl_2_. The organic layers were combined, dried over MgSO_4_, filtered, and concentrated under vacuum. The crude product was further purified by column chromatography on silica gel [Ethyl acetate/ether, 7:3 and then 6:4 + Et_3_N (1%)] affording **3** (9.3 g, 60%).

MS, ESI^+^ m/z: 525.2 [M+Na]^+^

R_f_: 0.4 [Cyclohexane/Et_2_O (1:1)]

^1^H NMR (400 MHz, CDCl_3_, 298 K): δ = 0.15, 0.15, 0.17 (3s, 27H, Me_3_Si), 3.49 (dt, *J*_H7-H8_ = 6.2 Hz, 2H, H-7), 3.64 (m, 1H, H-5), 3.79 (m, 4H, H-6, H-6', H-8', H-3), 3.80 (m, 1H, H-2), 3.89 (t, *J*_H4-H3_ = 9.1 Hz, 1H, H-4), 3.94-3.97 (dt, *J*_H8-H7’_ = 5.6 Hz, 1H, H-8), 4.66 (d, *J*_H1-H2_ = 1.9 Hz, 1H, H-1) ppm.

^13^C NMR (101 MHz, CDCl_3_, 298K): δ = 0.53, 0.83, 0.87 (9 C, 3 x Me_3_Si), 30.5 (1 C, C-9), 62.3 (1 C, C-6), 67.7 (1 C, C-8), 68.1 (1 C, C-4), 72.5 (1 C, C-3), 73.6 (1 C, C-2), 73.8 (1 C, C-5), 101.4 (1 C, C-1) ppm.

**2-bromoethyl 2,3,4-tri-*O*-trimethylsilyl-α-D-*manno*-hexodialdopyranoside (4)**

Compound **3** (200 mg, 0.43 mmol) was dissolved in freshly distilled DCM (9 mL). A solution of Dess-Martin periodinane in dichloromethane (0.3M, 2.17 mL, 0.65 mmol) was added. The reaction mixture was stirred at room temperature for 1h and then diluted with 9 mL of Et_2_O. To this mixture, a saturated solution of NaHCO_3_ (9 mL) and 1.56g of Na_2_S_2_O_3_ were added. The solution was stirred at room temperature for 30 min and was further diluted with 40 mL of Et_2_O. The aqueous layer was extracted with 4 x 20 mL of Et_2_O. The organic layers were combined, washed with 40 mL of water, dried with MgSO_4_, and concentrated to yield compound **4**, which was directly used for the next step.

R_f_: 0.34 [Cyclohexane/Et_2_O (1:1)]

**2-bromoethyl (E)-2,3,4-tri-*O*-trimethylsilyl-6,7-dideoxy-7-diethoxyphosphinyl-α-D-*manno*-hept-6-enopyranoside (5)**

A mixture of 60% NaH dispersion in oil (165 mg, 4.96 mmol) was suspended in THF (30 mL). Then, tetraethyl methylenediphosphonate (985 µL, 3.97 mmol) was added slowly drop by drop. The resulting reaction mixture was stirred at room temperature for 45 min before being added to compound **4** (995 mg, 1.99 mmol) dissolved in THF (15 mL). After stirring at room temperature for 1h, the reaction mixture was then diluted with 375 mL of CH_2_Cl_2_ and washed twice with brine (75 mL). The aqueous layer was further extracted with 2 x 250 mL of CH_2_Cl_2_. The organic layers were combined, dried with MgSO_4_, and concentrated. The resulting residue (brown oil) was purified by automated flash chromatography on silica gel (CH_2_Cl_2_/diethyl ether: 0 to 10% diethyl ether gradient) to obtain compound **5** as a light-yellow oil with a yield of 76% (956 mg, 1.5 mmol) over two steps.

R_f_: 0.28 [Cyclohexane/Et_2_O (2:8)]

MS, ESI^+^ m/z: 635.3 [M+H]^+^

^1^H NMR (400 MHz, CDCl_3_, 298K): δ = 0.14 (s, 18H, 2 x Me_3_Si), 0.17 (s, 9H, Me_3_Si), 1.33 (t, 6H, CH_3_-CH_2_-O, ^3^*J*_CH3-CH2_ = 7.0 Hz), 3.43 (t, 1H, ^3^*J*_H9-H8_ = 6.1 Hz, H-9), 3.44 (t, 1H, ^3^*J*_H9’-H8_ = 6.1 Hz, H-9’), 3.67 (t, ^3^*J*_H4-H3_ = 8.8 Hz, 1H, H-4), 3.74 (dt, ^3^*J*_H8’-H9_ = 6.1 Hz, ^2^J_H8’-H8_ = 11.4 Hz, H-8’), 3.76 (dd, ^3^*J*_H3-H2_ = 2.8 Hz, ^3^*J*_H3-H4_ = 8.4 Hz, H-3), 3.80 (dd, ^3^*J*_H2-H1_ = 1.9 Hz, ^3^*J*_H2-H3_ = 2.8 Hz, 1H, H-2), 3.90 (dt, ^3^*J*_H8’-H9_ = 6.1 Hz, ^2^*J*_H8’-H8_ = 11.4 Hz, H-8), 4.08-4.10 (dq, 4H, ^3^*J*_H-H_ = 7.0 Hz, CH_3_-CH_2_-O), 4.12 (m, 1H, H-5), 4.67 (d, ^3^*J*_H1-H2_ = 1.9 Hz, 1H, H-1), 6.04 (ddd, ^3^*J*_H7-H6_ = 17.2 Hz, ^2^J_H7-P_ = 21.9 Hz, ^4^*J*_H7-H5_ = 1.9 Hz, 1H, H-7), 6.84 (ddd, ^3^*J*_H6-H5_ = 4.2 Hz, ^3^*J*_H6-H7_ = 17.2 Hz, ^3^*J*_H6-P_ = 22.8 Hz, 1H, H-6) ppm.

^13^C NMR (101 MHz, CDCl_3_, 298K): δ = 0.4, 0.8, 0.9 (9 C, Me_3_Si), 16.5 (1 C, CH_3_-CH_2_-O), 16.6 (d, 1 C, ^3^*J*_C-P_ = 6.4 Hz, CH_3_-CH_2_-O), 30.5 (1 C, C-9), 61.8 (2 C, CH_3_-CH_2_-O), 67.7 (1 C, C-8), 71.5 (1 C, C-4), 72.8 (1 C, C-3), 73.4 (1 C, C-2), 73.8 (d, 1 C, ^3^*J*_C5-P_ = 20.7 Hz, C-5), 101.3 (1 C, C-1), 117.3 (d, 1 C, ^1^*J*C7-P = 190.0 Hz, C-7), 148.9 (d, 1 C, ^2^*J*_C6-P_ = 5.8 Hz, C-6) ppm.

^31^P NMR (162 MHz, CDCl_3_, 298K): δ = 18.4 ppm

**2-bromoethyl 6,7-dideoxy-7-diethoxyphosphinyl-α-D-*manno*-heptanopyranoside (6)**

Compound **5** (956 mg, 1.5 mmol) was dissolved in methanol (22 mL). 10% palladium on charcoal (143 mg, 15% by weight) was added. Triethylsilane (2.4 mL, 15 mmol) was then added dropwise over 2h. The reaction mixture was filtered through celite, and the filtrate was evaporated to lead to compound **6** with a yield of 99% (632 mg, 1.5 mmol).

R_f_: 0.28 [CH_2_Cl_2_/MeOH (95:5)]

MS, ESI^+^ m/z: 422.9 [M+H]^+^

^1^H NMR (600 MHz, MeOD, 298K): δ = 1.38 (t, 6H, CH_3_-CH_2_-O, ^3^*J*_CH3-CH2_ = 6.6 Hz), 1.65-1.75 (m, 1H, H-6), 1.84 (dddd, 1H, ^3^*J*_H7-H6_ = 3.8 Hz, ^3^*J*_H7-H6’_ = 10.6 Hz, ^2^*J*_H7-H7’_ = 15.5 Hz, ^2^*J*_H7-P_ = 26.1 Hz, H-7), 1.98-2.05 (m, 1H, H-7’), 2.05-2.14 (m, 1H, H-6’), 3.45 (t, ^3^*J*_H4-H3_ = 9.4 Hz, 1H, H-4), 3.56 (t, 1H, ^3^*J*_H9-H8’_ = ^3^*J*_H9-H8_ = 5.5 Hz, H-9), 3.56 (“t”, 1H, ^3^*J*_H9’-H8_ = 5.5 Hz, ^3^*J*_H9’-H8’_ = 5.4 Hz, H-9’), 3.59 ( td, 1H, ^3^*J*_H5-H6_ = 2.6 Hz, ^3^*J*_H5-H4_ = 9.4 Hz, H-5), 3.67 (dd, 1H, ^3^*J*_H3-H2_ = 3.4 Hz, ^3^*J*_H3-H4_ = 9.4 Hz, H-3), 3.80 ( dt, ^3^*J*_H8-H9’_ = 5.5 Hz, ^2^*J*_H8-H8’_ = 11.4 Hz, 1H, H-8), 3.88 (dd, ^3^*J*_H2-H1_ = 1.2 Hz, ^3^*J*_H2-H3_ = 3.6 Hz, 1H, H-2), 3.97 ( ddd, ^3^*J*_H8’-H9_ = 5.3 Hz, ^3^*J*_H8’-H9’_ = 6.4 Hz, ^2^*J*_H8’-H8_ = 11.4 Hz, 1H, H-8’), 4.10-4.20 (m, 4H, CH_3_-CH_2_-O), 4.78 (d, ^3^*J*_H1-H2_ = 1.6 Hz, 1H, H-1) ppm.

^13^C NMR (150 MHz, MeOD, 298K): δ = 16.7 (d, ^3^*J*_C-P_ = 5.9 Hz, 1C, CH_3_-CH_2_-O), 22.0 (d, ^1^*J*_C7-P_ = 141.9 Hz, 1C, C-7), 25.6 (d, ^2^*J*_C6-P_ = 3.8 Hz, 1C, C-6), 31.7 (1C, C-9), 63.2 (2C, CH_3_-CH_2_-O), 68.9 (1C, C-8), 71.7 (1C, C-4), 72,0 (1C, C-2), 72,4 (1C, C-3), 73,4 (d, 1C, ^3^*J*_C5-P_ = 7.2 Hz, C-5), 101.8 (1C, C-1) ppm.

^31^P NMR (243 MHz, MeOD, 298K): δ = 33.8 ppm.

**2-(phtalimido)ethyl 6,7-dideoxy-7-diethoxyphosphinyl-α-D-*manno*-heptanopyranoside (7)**

Compound **6** (212 mg, 0.5mmol) was dissolved in anhydrous DMF (2 mL). Potassium phthalimide (140 mg, 0.76mmol) was added. The suspension was stirred for 22h at 60°C and then evaporated. The resulting residue was purified by silica gel chromatography (AcOEt/MeOH: 10 to 40% MeOH gradient) to yield compound **7** as a colorless oil with a 74% yield (182 mg, 0.37mmol).

R_f_: 0.20 [AcOEt/MeOH (9:1)]

MS, ESI^+^ m/z: 487.8 [M+H]^+^

^1^H NMR (600 MHz, MeOD, 298K): δ = 1.39 (t, 6H, CH_3_-CH_2_-O, ^3^*J*_CH3-CH2_ = 7.2 Hz,), 1.59-1.70 (m, 1H, H-6), 1.71-1.79 (m, 1H, H-7), 1.81-2.07 (m, 1H, H-7’ and H-6’), 3.35 ( dt, 1H, ^3^*J*_H5-H6_ = 2,1 Hz, ^3^*J*_H5-H4_ = 9.4 Hz, H-5), 3.44 (t, 1H, ^3^*J*_H4-H3_ = 9.4 Hz , H-4), 3.59 (dd, 1H, ^3^*J*_H3-H2_ = 3.4 Hz, ^3^*J*_H3-H4_ = 9.4 Hz, H-3), 3,75 ( ddd, ^3^*J*_H8-H9’_ = 4.6 Hz, ^3^*J*_H8-H9_ = 5.3 Hz, ^2^*J*_H8-H8’_ = 10.0 Hz, 1H, H-8), 3.78 (dd, ^3^*J*_H2-H1_ = 1.5 Hz, ^3^*J*_H2-H3_ = 3.4 Hz, 1H, H-2), 3.90 ( ddd, ^3^*J*_H9-H8’_ = 4.6 Hz, ^3^*J*_H9-H8_ = 5.3 Hz, ^2^*J*_H9-H9’_ = 13.5 Hz, 1H, H-9), 3,96 ( ddd, ^3^*J*_H8’-H9_ =4.6 Hz, ^3^*J*_H8’-H9’_ =7.3 Hz, ^2^*J*_H8’-H8_ = 10.0 Hz, 1H, H-8’), 4.02 ( ddd, ^3^*J*_H9’-H8’_ = 4.6 Hz, ^3^*J*_H9’-H8_ = 7.3 Hz, ^2^*J*_H9’-H9_ = 13.5 Hz, 1H, H-9’), 4.12-4.19 (m, 4H, CH_3_-CH_2_-O), 4.80 (d, ^3^*J*_H1-H2_ = 1.5 Hz, 1H, H-1), 7.85-7.88 (m, 2H, Ar), 7.90-7.93 (m, 2H, Ar) ppm.

^13^C NMR (150 MHz, MeOD, 298K): δ = 16.7 (d, ^3^*J*_C-P_ = 5.7 Hz, 2C, CH_3_-CH_2_-O), 22.0 (d, ^1^*J*_C7-P_ = 141.0 Hz, 1C, C-7), 25.3 (d, ^2^*J*_C6-P_ = 3.9 Hz, 1C, C-6), 38.4 (1C, C-9), 63.2 (m, 2C, CH_3_-CH_2_-O), 64.9 (1C, C-8), 71.6 (1C, C-4), 71.9 (1C, C-3), 72.3 (1C, C-2), 73.4 (d, 1C, ^3^*J*_C-P_ = 17.6 Hz, C-5), 101.2 (1C, C-1), 124.2 (2C, Ar), 133.3 (2C, Ar), 135.5 (2C, Ar), 169.7 (2C, C=O) ppm.

^31^P NMR (243 MHz, MeOD, 298K): δ = 33.63 ppm.

**2-(phtalimido)ethyl 2,3,4-tri-O-trimethylsilyl-6,7-dideoxy-7-diethoxyphosphinyl-α-D-*manno*-heptanopyranoside (8)**

Compound **7** (107 mg, 0.22 mmol) was dissolved in anhydrous dichloromethane DCM (635 µL) along with triethylamine (890 µL, 6.59 mmol). Trimethylsilyl chloride (167 µL, 1.32 mmol) was added dropwise at 0°C. The solution was stirred at room temperature for 16h and then evaporated. The resulting pink residue was dissolved in Et_2_O and filtered through celite. The filtrate was concentrated to yield compound **8** directly used for the next step (154 mg, 0.22 mmol).

R_f_: 0.74 [AcOEt/MeOH (9:1)]

**2-aminoethyl 6,7-dideoxy-7-diethoxyphosphinyl-α-D-*manno*-heptanopyranoside (9)**

To a solution of **8** (154 mg, 0.22 mmol) in anhydrous CH_3_CN (3 mL) was successively added pyridine (204 µL, 2.5 mmol) and trimethylsilyl bromide (290 µL, 2.2 mmol). The reaction mixture was stirred for 6h at room temperature and solvents were then evaporated. The residue was dissolved in MeOH and excess of pyridinium salts were filtered out.

A solution of hydrazine monohydrate (85 µL, 1.75 mmol) in methanol (3 mL) was added to the previously obtained residue. The reaction mixture was stirred at room temperature for 16h. The solvents were evaporated, and the resulting residue was purified by reversed-phase silica gel chromatography (Water) to obtain compound **9** as a white solid with a 65% yield (42 mg, 0.14 mmol).

R_f_: 0.22 [Isopropanol/NH_4_OH/H_2_O (5:3:2)]

MS, ESI^+^ m/z: 302.2 [M+H]^+^

^1^H NMR (600 MHz, D_2_O, 298K): δ = 1.45-163 (m, 1H, H-7’), 1.66-1.72 (m, 2H, H-6’ and H-7), 1.75-2.06 (m, 1H, H-6), 3.22 (ddd, 1H, ^3^*J*_H9-H8’_ = 4,1 Hz, ^3^*J*_H9-H8_ = 6.3 Hz, ^2^*J*_H9-H9’_ = 13.5 Hz, H-9), 3.25 (ddd, 1H, ^3^*J*_H9’-H8_ = 4.0 Hz, ^3^*J*_H9’-H8’_ = 6.3 Hz, ^2^*J*_H9’-H9_ = 13.5 Hz, H-9’), 3.54 (dd, 1H, ^3^*J*_H4-H3_ = 9.2 Hz , ^3^*J*_H4-H5_ = 9.6 Hz, H-4), 3.58 ( ddd, 1H, ^3^*J*_H5-H6_ = 2.5 Hz, ^3^*J*_H5-H4_ = 9.6 Hz, ^3^*J*_H5-H6’_ = 8.4 Hz, H-5), 3.69 (ddd, ^3^*J*_H8-H9’_ = 4.0 Hz, ^3^*J*_H8-H9_ = 6.3 Hz, ^2^*J*_H8-H8’_ = 11.4 Hz, 1H, H-8), 3.80 (dd, 1H, ^3^*J*_H3-H2_ = 3.5 Hz, ^3^*J*_H3-H4_ = 9.2 Hz, H-3), 3.98 ( ddd, ^3^*J*_H8’-H9_ = 4.1 Hz, ^3^*J*_H8’-H9’_ = 6.3 Hz, ^2^*J*_H8’-H8_ = 11.4 Hz, 1H, H-8’), 3.98 (dd, ^3^J_H2-H1_ = 1.7 Hz, ^3^*J*_H2-H3_ = 3.5 Hz, 1H, H-2), 4.85 (d, ^3^*J*_H1-H2_ = 1.7 Hz, 1H, H-1) ppm.

^13^C NMR (150 MHz, D_2_O, 298K): δ = 25.8 (d, ^1^*J*_C7-P_ = 130.7 Hz, 1C, C-7), 27.1 (d, ^2^*J*_C6-P_ = 1.9 Hz, 1C, C-6), 40.3 (1C, C-9), 64.7 (1C, C-8), 71.2 (1C, C-2), 71.4 (1C, C-4), 71.7 (1C, C-3), 74.2 (d, 1C, ^3^*J*_C5-P_ = 14.6 Hz, C-5), 101.1 (1C, C-1) ppm.

^31^P NMR (243 MHz, D_2_O, 298K): δ = 25.5 ppm.

[α]_D_^20^ = +40.18° (c=5.6 x 10^-3^M/H_2_O)

**2-((4-ethoxy-2,3-dioxocyclobut-1-enyl)amino)ethyl 6,7-dideoxy-7-diethoxyphosphinyl-α-D-*manno*-heptanopyranoside (AMFA2)**

Compound **9** (23 mg, 0.076mmol) was dissolved in a mixture of EtOH/H_2_O (2:1) (680 µL). Diethyl squarate (11.3 µL, 0.076mmol) was added, followed by the addition of triethylamine (21.2 µL, 0.076 mmol) to reach a pH of 9. The reaction mixture was stirred at room temperature for 30 min and then evaporated. The resulting residue was purified by preparative HPLC (H_2_O/acetonitrile: 0 to 15% acetonitrile gradient) to yield **AMFA2** as a yellow solid with a 50% yield.

R_f_: 0.71 [MeOH/H_2_O (8:2)]

MS, ESI^+^ m/z: 426.2 [M+H]^+^

^1^H NMR (400 MHz, D_2_O, 353K): δ = 1.96 (t, 3H, ^3^*J*_H-H_ = 7.2 Hz, CH_3_-CH_2_-O), 1.69-1.98 (m, 1H, H-7’), 1.98-2.21 (m, 2H, H-6’ and H-7), 2.38-2.53 (m, 1H, H-6), 3.95 (dt, 1H, ^3^*J*_H5-H6_ = 2.8 Hz, ^3^J_H5-H4_ = ^3^*J*_H5-H6’_ = 9.2 Hz, H-5), 4.05 (t, 1H, ^3^*J* = 9.2 Hz, H-4), 4.12-4.37 (m, 5H, H-8, H-8’, H-9, H-9’, H-3), 4.39 (dd, 1H, ^3^*J*_H2-H1_ = 1.6 Hz, ^3^*J*_H2-H3_ = 3.2 Hz, H-2), 5.26 (q, 2H, ^3^*J*_H-H_ = 7.2 Hz, CH_3_-CH_2_-O), 5.33 (d, 1H, ^3^*J*_H1-H2_ = 1.6 Hz, H-1) ppm.

^13^C NMR (101 MHz, D_2_O, 353K): δ = 24.5 (d, ^1^*J*_C7-P_ = 135.3 Hz, 1C, C-7), 25.9 (s, 1C, C-6), 43.9 (1C, C-9), 67.51 (1C, C-8), 70.9 (2C, C-2, C-4), 71.4 (2C, CH_3_-CH_2_-O, C-3), 73,4 (d, 1C, ^3^*J*_C5-P_ = 16.3 Hz, C-5), 100.3 (1C, C-1), 174.7 (1C, C=C-NH), 178.1 (1C, C=C-OEt), 184.2 (1C, C=O), 189.0 (1C, C=O) ppm.

^31^P NMR (162 MHz, D_2_O, 353K): δ = 26.0 ppm.

**
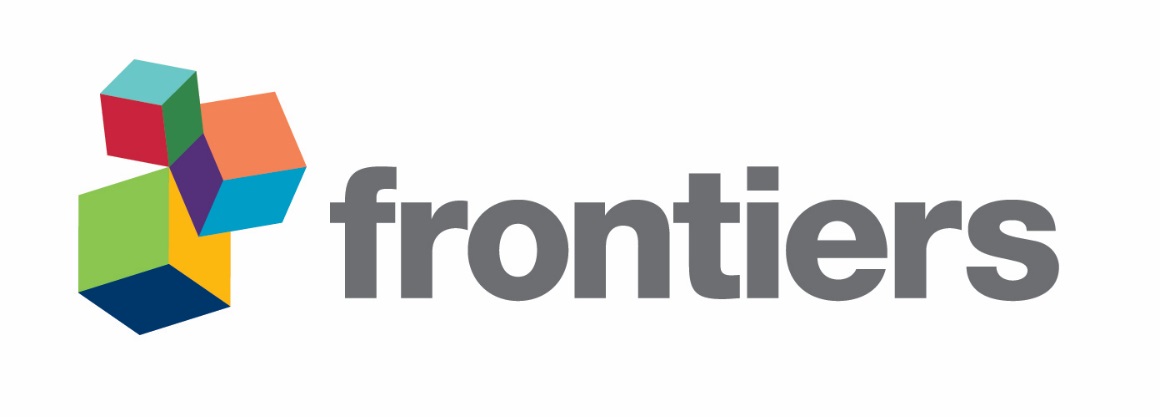
**
