# Supplementary figures and images for: Engineered therapeutic antibodies with mannose 6-phosphate analogues as a tool to degrade extracellular proteins
Source: Front Immunol. 2024 Mar 12;15:1273280. doi: 10.3389/fimmu.2024.1273280 (PMC10964947; doi:10.3389/fimmu.2024.1273280)

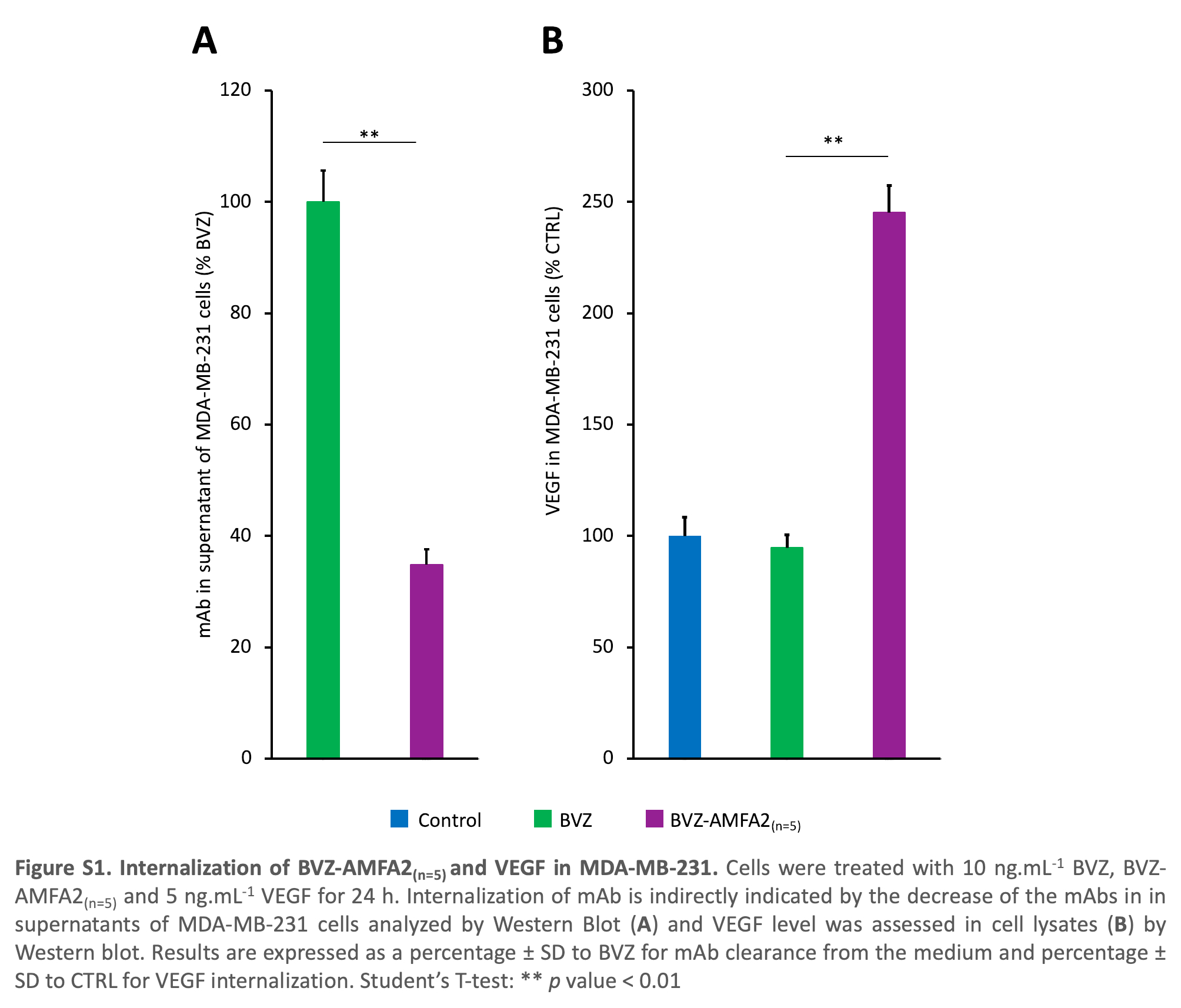

Supplement: Supplementary file 2 [file Image_1.jpg]

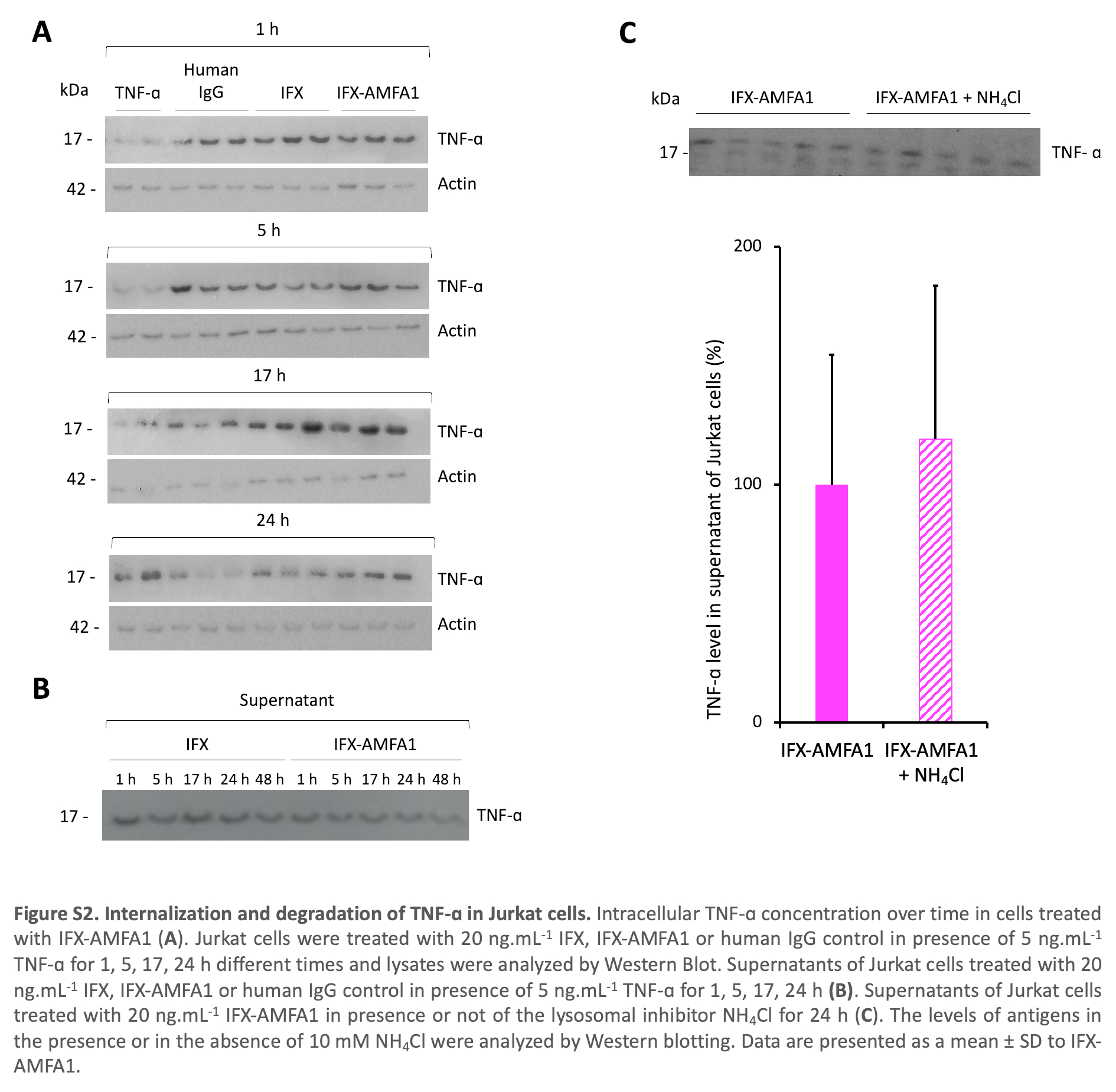

Supplement: Supplementary file 3 [file Image_2.jpg]

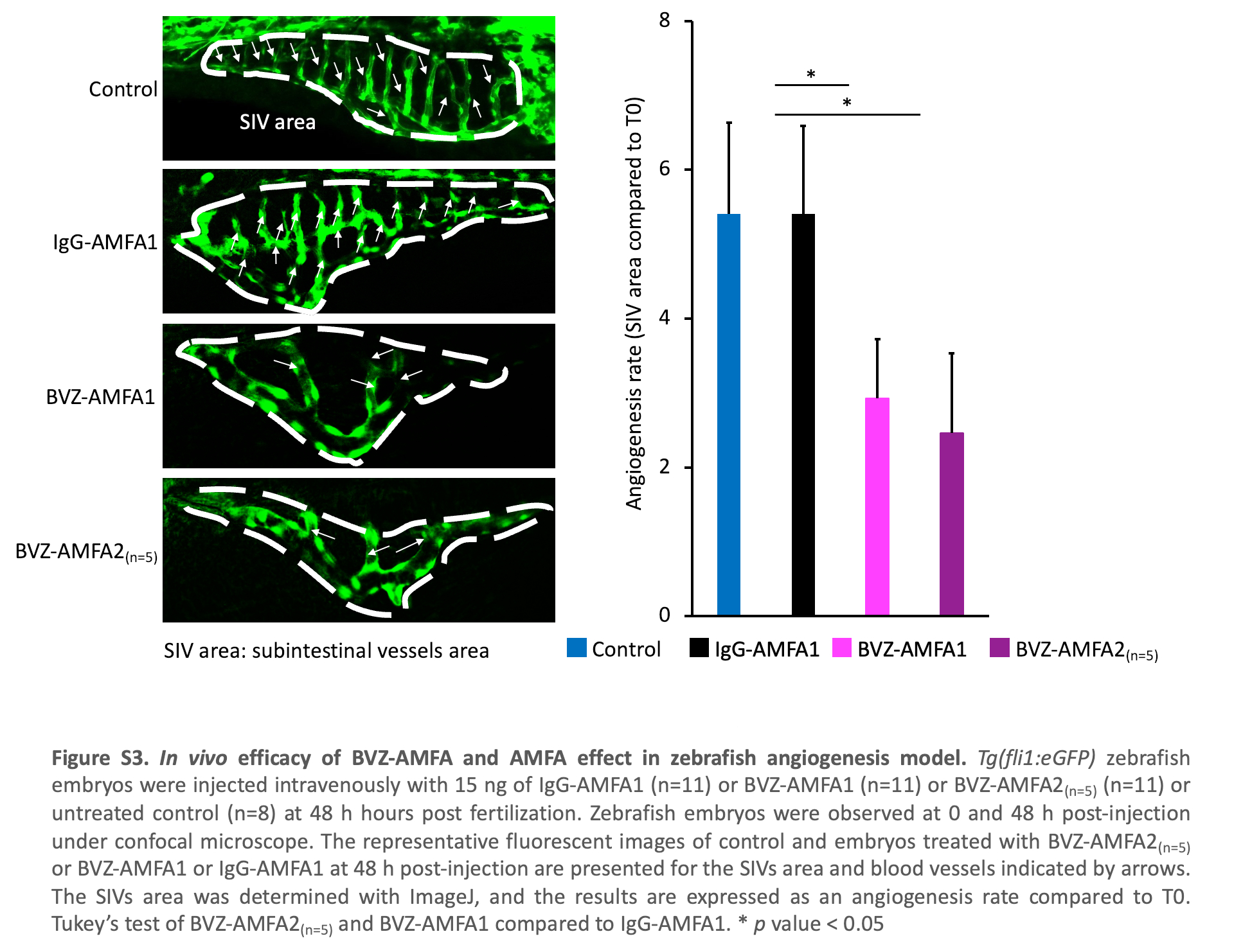

Supplement: Supplementary file 4 [file Image_3.jpg]
